# Spindle Formation in the Mouse Embryo Requires Plk4 in the Absence of Centrioles

**DOI:** 10.1016/j.devcel.2013.09.029

**Published:** 2013-12-09

**Authors:** Paula A. Coelho, Leah Bury, Bedra Sharif, Maria G. Riparbelli, Jingyan Fu, Giuliano Callaini, David M. Glover, Magdalena Zernicka-Goetz

**Affiliations:** 1 Cancer Research UK Cell Cycle Genetics Group, Department of Genetics, University of Cambridge, Cambridge CB2 3EH, UK; 2 Pluripotency and Differentiation in Early Mouse Development Group, Gurdon Institute and Department of Physiology, Development and Neuroscience, University of Cambridge, Cambridge CB2 1QN, UK; 3 Department of Evolutionary Biology, University of Siena, Via A. Moro 41-53100, Siena, Italy

## Abstract

During the first five rounds of cell division in the mouse embryo, spindles assemble in the absence of centrioles. Spindle formation initiates around chromosomes, but the microtubule nucleating process remains unclear. Here we demonstrate that Plk4, a protein kinase known as a master regulator of centriole formation, is also essential for spindle assembly in the absence of centrioles. Depletion of maternal Plk4 prevents nucleation and growth of microtubules and results in monopolar spindle formation. This leads to cytokinesis failure and, consequently, developmental arrest. We show that Plk4 function depends on its kinase activity and its partner protein, Cep152. Moreover, tethering Cep152 to cellular membranes sequesters Plk4 and is sufficient to trigger spindle assembly from ectopic membranous sites. Thus, the Plk4-Cep152 complex has an unexpected role in promoting microtubule nucleation in the vicinity of chromosomes to mediate bipolar spindle formation in the absence of centrioles.

## Highlights

• Plk4 is at acentriolar MTOCs and spindle poles in mouse embryos
• Plk4 is essential for acentriolar spindle assembly
• Depletion of maternal Plk4 prevents normal nucleation and growth of microtubules
• Plk4 MT-nucleating function depends on its kinase activity and its partner, Cep152

## Teaser

During the first five rounds of division in the mouse embryo, spindles assemble in the absence of centrioles. Here, Coelho et al. show that protein kinase Plk4, the master regulator of centriole formation, and its partner protein Cep152 are also essential for assembly of acentriolar spindles in the mouse embryo.

## Introduction

There are two main pathways for the assembly of meiotic or mitotic spindles. The first is the “search and capture” mechanism, whereby centrosome nucleated microtubules (MTs) make contact with and are stabilized by kinetochores (Kirschner and Mitchison, 1986). This pathway depends on centrosomes as the major centers for organizing MTs. Typically centrosomes comprise a pair of centrioles, surrounded by pericentriolar material (PCM; Nigg and Stearns, 2011). Plk4, Polo-like kinase family member, has been established as a conserved key regulator of centriole formation (Bettencourt-Dias et al., 2005; Habedanck et al., 2005). Loss of Plk4 prevents centriole formation and its overexpression leads to de novo formation of centrioles in both unfertilized *Drosophila* eggs as well as in *Xenopus*-activated oocytes and extracts (Rodrigues-Martins et al., 2007; Eckerdt et al., 2011). To mediate centriole formation, Plk4 requires Cep152/Asterless to recruit PCM and permit spindle assembly (Blachon et al., 2008; Bonaccorsi et al., 1998; Cizmecioglu et al., 2010; Dzhindzhev et al., 2010; Hatch et al., 2010; Varmark et al., 2007).

In the second pathway, MTs are organized around chromosomes and then sort into bipolar antiparallel arrays (Meunier and Vernos, 2012). Chromosome-mediated spindle assembly is crucial for female meiosis in many species because centrioles are eliminated during oogenesis (Delattre and Gönczy, 2004). The chromosome-mediated pathway also permits spindle formation in cultured cells after centrosome elimination (Hinchcliffe et al., 2001; Khodjakov and Rieder, 2001; Mahoney et al., 2006; Rieder et al., 2001) and can be observed when MTs are depolymerized and then allowed to regrow (Khodjakov et al., 2000; Torosantucci et al., 2008). In the latter case, MTs are nucleated by centrosomes and around chromosomes, indicating that both centrosome- and chromosome-mediated spindle formation mechanisms can exist side-by-side (Meunier and Vernos, 2012).

The maturing mouse oocyte and preimplantation mouse embryo provide naturally occurring acentriolar cells for the first 4 days of development. This is because mouse sperm does not contribute basal bodies at fertilization and therefore all cell divisions take place in the absence of centrioles until the blastocyst stage (Calarco-Gillam et al., 1983; Courtois et al., 2012; Gueth-Hallonet et al., 1993; Hiraoka et al., 1989). In the absence of centrioles, microtubule organizing centers (MTOCs) are dispersed throughout the cytoplasm and then coalesce at the poles of the spindle as it forms (Calarco, 2000; Delattre and Gönczy, 2004; Dumont et al., 2007; Schuh and Ellenberg, 2007).

Here, we show that Plk4 is present in the MTOCs from the earliest stages of mouse embryonic development but it does not drive de novo centriole formation. Instead, Plk4 plays a critical function in partnership with Cep152 to mediate normal MT nucleation and growth to enable acentrosomal spindle assembly. These results unveil a role for Plk4 and Cep152 that is independent of centriole duplication.

## Results

### Plk4 Associates with MTOCs but Does Not Drive Centriole Formation in the Early Mouse Embryo

**Figure 1:**
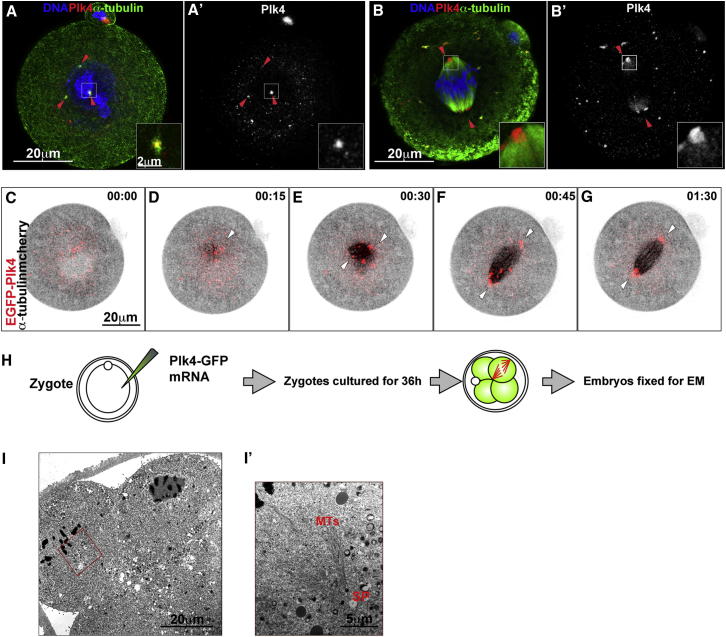
Plk4 Associates with MTOCs throughout the Cell Cycle in the Absence of Centrioles (A and B) Mouse zygotes stained to reveal α-tubulin (green), Plk4 (red and monochrome, A′ and B′), and DNA (blue). Plk4 (red arrowheads) associates with cytoplasmic MTOCs in interphase and spindle poles in mitosis (B). (C–G) Time-lapse series of zygotes expressing α-tubulin-mcherry (inverted black) and EGFP-Plk4 (red) showing multiple interphase MTOC-associated Plk4 bodies that coalesce at spindle pole in mitosis. Time is in hr:min. See also Movie S1. (H) Schematic of protocol for Plk4 overexpression from zygote stage prior to electron microscopy at four-cell stage. (I) Electron micrographs of a four-cell embryo that has had elevated Plk4-GFP from the zygote stage. (I′) Magnified inset outlined in red box in (I): microtubules (MT) and spindle poles (SP) devoid of centrioles are indicated. See also Figure S1 and Movie S1.

We first considered whether the absence of centrioles in the mouse embryo could be due to the lack of Plk4, the master regulator of the centriole formation pathway (Bettencourt-Dias et al., 2005; Habedanck et al., 2005). We found that Plk4 transcripts are present in preimplantation development and are already abundant in the oocyte and zygote, indicative of their maternal contribution (Wang et al., 2004), but to determine whether Plk4 protein is also present at these early stages, we generated an anti-Plk4 antibody (Figures S1A–S1E available online). This antibody specifically interacted with Plk4 (Figures S1F and S1G) and identified tissue culture cell centrioles by structured illumination microscopy (Figures S1H and S1I). The antibody also allowed us to reveal the presence of Plk4 in the MTOCs of the mouse zygote at interphase and at the acentriolar spindle poles as the spindle forms (arrowheads, Figures 1A and 1B; n = 20). The presence of Plk4 at the spindle poles in the mouse zygote was unexpected because Plk4 is a major regulator of centriole formation and yet centrioles were reported to be absent until the blastocyst stage (Calarco-Gillam et al., 1983). In agreement, we confirmed that colocalization of centriole-associated proteins, including Centrin 2, Sas6, and γ-tubulin, does not occur until the blastocyst stage (Figure S1J). Thus, despite the presence of Plk4, centrioles do not form in the mouse embryo until the blastocyst stage.

To follow the dynamics of Plk4 during spindle formation in vivo, we injected mRNAs for EGFP-Plk4 and α-tubulin-mcherry into the zygote (Figures 1C–1G). This revealed that EGFP-Plk4 showed a similar pattern of subcellular localization to the endogenous protein. During interphase, Plk4 associated with discrete punctate cytoplasmic structures that become concentrated at MT nucleation sites in the vicinity of chromatin at the time of nuclear envelope breakdown (NEBD; Figures 1C and 1D). As the spindle formed, the Plk4-containing centers coalesced at the spindle poles (Figures 1E–1G; Movie S1) in a pattern that resembles the behavior of MTOCs during meiotic and mitotic divisions (Calarco-Gillam et al., 1983; Courtois et al., 2012; Gueth-Hallonet et al., 1993; Hiraoka et al., 1989; Maro et al., 1985). These time-lapse movies revealed no variation in timing of M-phase progression of the first cleavage between Plk4-GFP injected (n = 30; 127.9 ± 11.2 min) and α-tubulin-mcherry injected control zygotes (n = 22; 136.3 ± 16.0 min). This overexpression of Plk4 did not lead to formation of centrioles; examination of the spindle poles of dividing cells in serial EM sections of embryos expressing elevated Plk4-GFP that reached the four-cell stage did not reveal any centrioles (Figures 1I and 1I′). These results indicate that Plk4 is present in the early mouse embryo but its presence is insufficient to drive centriole formation.

### Plk4 Is Required for MT Nucleation and Spindle Assembly

**Figure 2:**
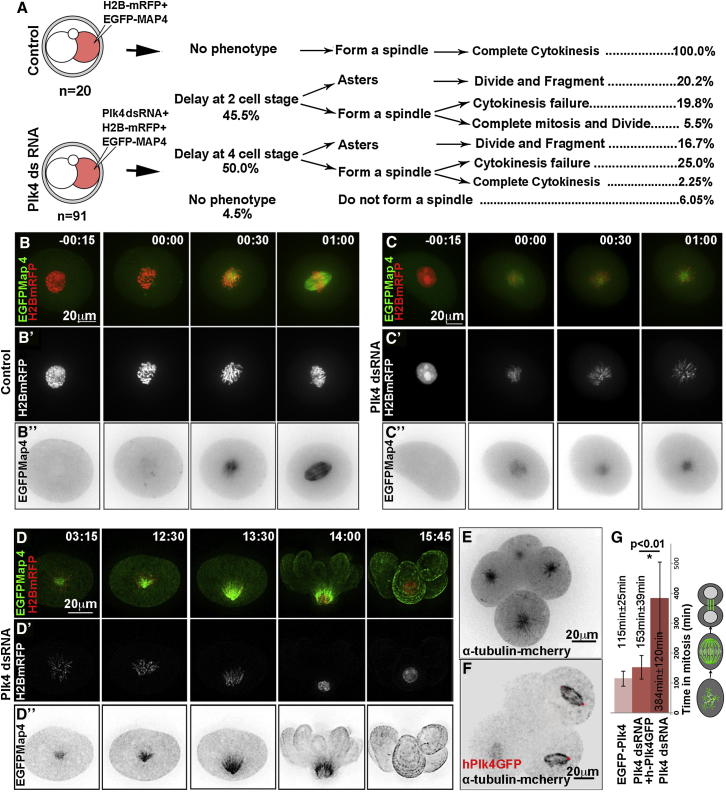
Depletion of Plk4 Leads to Monoaster Formation and Mitotic Delay (A) Schematic for injection of single two-cell stage blastomere with Plk4 dsRNA, together with mRNAs for labeled histone H2B and MAP4 indicating proportions of embryos showing abnormalities at subsequent stages. (B) Control two-cell stage embryo with one cell expressing histone H2B-mRFP (red, merged; white; B′) and EGFP-MAP4 (green, merged; inverted/black; B″). Time (hr:min) is in relation to NEBD, 00:00. See also Movie S2. (C and D) Plk4 dsRNA injected two-cell blastomere, labeled as in (B). MT nucleation is severely reduced and a single aster eventually forms (C). Monoasters move toward the cell cortex, and cells undergo highly abnormal cytokinesis (D). (E–G) Rescue of Plk4 knockdown phenotype (shown at eight-cell stage in E), by coinjection of human PLK4-GFP (red, F). MTs revealed by α-tubulin-mcherry, inverted/black. Time for bipolar spindle assembly following Plk4 dsRNAi is increased in cells that evade monoaster formation. This is greatly restored to levels similar to controls by injection of human PLK4-GFP mRNA (G) and are statistically significant between these two groups (^∗^p < 0.01). Error bars indicate SD of average. See also Movie S2 and Figure S2.

The finding that Plk4 is present in mouse embryos that lack centrioles raised the question of whether Plk4 plays any functional role at these early developmental stages. To address this, we followed the development of embryos depleted of maternal and zygotic Plk4 transcripts by RNAi (n = 91; Figure 2; Figures S2A and S2B). To follow the behavior of MTs and chromosomes using time-lapse microscopy, we coinjected mRNA for EGFP-Map4 and Histone H2B-mRFP. The Plk4 dsRNA was injected into one cell at the two-cell stage so that the untreated half of the embryo could serve as a control for developmental progression. A parallel set of control embryos were also injected at the two-cell stage with EGFP-Map4 and Histone H2B-mRFP (n = 20). In contrast to the control embryos, which developed without mitotic abnormalities, the great majority (95.5%, n = 87) of Plk4-depleted embryos either developed mitotic defects during the division cycle in which Plk4 was depleted or in the subsequent cycle (Figure 2A). The earliest cellular phenotype observed was in spindle assembly. In control cells, MT nucleation was visible in the vicinity of chromosomes soon after chromosome condensation and within 30 min of NEBD (n = 20; 100%; Figure 2B; Movie S2). Thirty minutes later, MTs became organized into a bipolar array and chromosomes congressed onto the metaphase plate. In contrast, Plk4-depleted cells showed diminution of MT nucleation around chromosomes, typically leading to the formation of weak monoastral structures by 60 min after NEBD (n = 42; 45.5%; Figure 2C; Movie S2). Irrespective of whether Plk4-depleted cells were delayed with monopolar spindles in the cycle immediately following Plk4 RNAi (n = 42; Figures 2C and 2D; Movie S2) or in the following cycle (n = 46; Figures S2C and S2D; Movie S2), mitosis resolved in similar ways. Whereas in control cells, mitosis and cytokinesis was completed within 2 hr (Figure S2C; Movie S2), Plk4-depleted cells could be delayed in mitosis for up to 11 hr (n = 19; Figure 2D; Figure S2D; mean delay, 384 ± 120 min; Movie S2). During this time, the monoasters relocated toward the cell cortex before the cells attempted cytokinesis that was highly abnormal and generated multiple anucleate fragments (Figure 2D, 14 hr time point; and Movie S2).

To ensure that the consequence of Plk4 depletion was not an off-target response, we examined whether the Plk4-RNAi phenotype could be rescued by coinjection of mRNA for human Plk4-GFP that is resistant to dsRNA directed against the mouse gene (n = 33). Using identical conditions of knockdown, we found that all phenotypes of Plk4-depletion were rescued by provision of human Plk4 (Figures 2E–2G). Specifically, monoasters (Figure 2E; Movie S2) were replaced by bipolar spindles (Figure 2F; Movie S2), the normal timing of mitotic progression was restored (Figure 2G), and the proportion of embryos showing cytokinesis defects decreased from 81.7% to 9.2%, indicating that the mitotic abnormalities upon Plk4-RNAi are specific to the depletion of Plk4. Together, these results suggest that despite the absence of centrioles, Plk4 has a functional role to mediate correct bipolar spindle formation in the mouse embryo. This early function of Plk4 would have been missed in studies of Plk4^−/−^ embryos (Hudson et al., 2001), because their heterozygous mothers provide sufficient Plk4 to support the first days of development.

### Plk4 Regulates MT Dynamics and Growth

**Figure 3:**
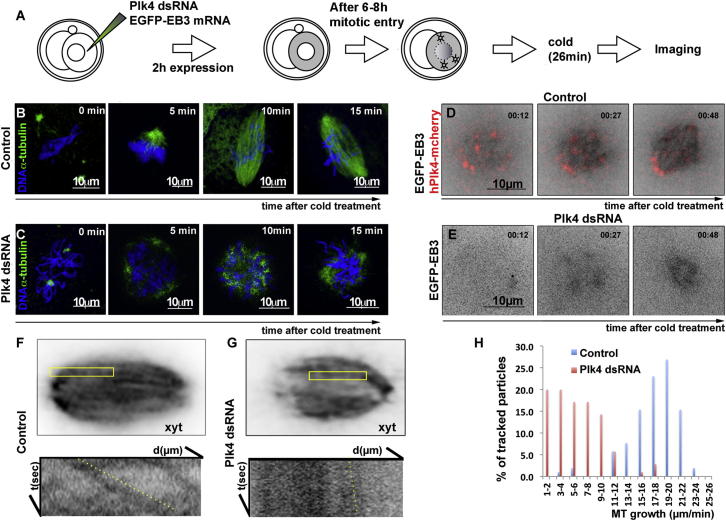
Plk4 Is Required for MT Dynamics and Growth (A) Schematic representation of MT regrowth assay. A single two-cell stage blastomere was injected with mRNA for EGFP-EB3 either with or without (control) Plk4 dsRNA. EGFP-EB3 mRNA expression begins within 2 hr and at least 6 additional hours are required for blastomeres to enter mitosis during which period, Plk4 is depleted. Upon entry into mitosis, embryos were subjected to cold treatment for 26 min, after which they were either fixed for immunostaining or imaged alive to follow dynamics of MT regrowth. (B and C) Two-cell embryos fixed at indicated times following release from cold treatment and stained for DNA (blue) and α-tubulin (green). The cold treatment completely depolymerized MTs (0 min) that regrew into a bipolar mitotic spindle within 10 min. In contrast, MT repolymerization was severely delayed in Plk4 dsRNAi-depleted blastomeres. (D and E) In vivo time-lapse microscopy following release from cold treatment. In control two-cell stage embryos (D) in which one blastomere was injected with mRNA for EGFP-EB3 and Plk4-mcherry, regrowth of MTs begins around sites of major MTOCs, in the vicinity of Plk4-mcherry fluorescence; bipolar spindle formation is seen by 48 min. In Plk4-RNAi-treated embryos (E), MT regrowth after cold exposure is impaired. See also Movie S3. (F and G) Time projection of metaphase spindles depicted by EGFP-EB3 expression. MT growth at plus tips shown on kymographs of EGFP-EB3 signals in control (F′) and Plk4-depleted (G′) blastomeres within the indicated areas of the spindles. The average pixel intensity projection of a metaphase spindle is analyzed for control (n = 12) and Plk4 RNAi blastomeres (n = 8). MT growth was determined from the slopes of EGFP-EB3 (yellow lines on kymographs). (H) MT growth rate distribution in control (average = 18.2 ± 3.5 μm/min; n = 56) and Plk4 RNAi cells (5.9 ± 4.04 μm/min; n = 35).

Because the above results suggest that Plk4 might be critical for MT functions, we next sought to determine the consequences of Plk4 depletion upon the dynamics of MT regrowth following depolymerization. To this end, we first depleted Plk4, as above, and coinjected embryos with mRNA for EGFP-tagged EB3 to mark MT plus ends (Figure 3A). Plk4-depleted and control embryos were subjected to 26 min cold treatment to depolymerize MTs after which MT regrowth was scored in both live (n = 20) and fixed (n = 35) embryos (Figures 3A–3E). In control embryos, we observed formation of MTs at the MTOCs that moved into the interstitial spaces between chromosomes (n = 10; Figure 3B; 0 and 5 min). These MTs rapidly sorted into a bipolar array and the MTOCs aggregated at the spindle poles (Figure 3B; 10 and 15 min). The areas of maximal EB3 signal clustered around Plk4 foci (Figure 3D, 00:27 min; Movie S3). In contrast, in Plk4-depleted embryos, the density of MTs was reduced and they tended to nucleate around the periphery of the ball of chromosomes (n = 10; Figures 3C and 5 and 10 min; Figure 3E, 00:27 min; Movie S3). These results suggest that Plk4 is required for robust foci of MTs to form in the vicinity of the condensed chromosomes.

In a second series of experiments, we omitted the MT depolymerization step and utilized EB3 fluorescence to record plus-end MT dynamics by time-lapse microscopy (n = 20; Figures 3F and 3G). Kymographs of the recordings revealed that in Plk4-depleted cells, growth of the EB3-associated MT plus tips toward the chromosomes was severely diminished (18.2 ± 3.5 μm/min in control versus 5.9 ± 4.04 μm/min in Plk4-depleted cells; Figures 3F and 3H). Taken together, these results indicate that Plk4 is required to establish the density and dynamicity of MTs in spindle assembly in the absence of centrioles.

### Spindle Assembly in Mouse Embryos Relies on Plk4 Kinase Activity

**Figure 4:**
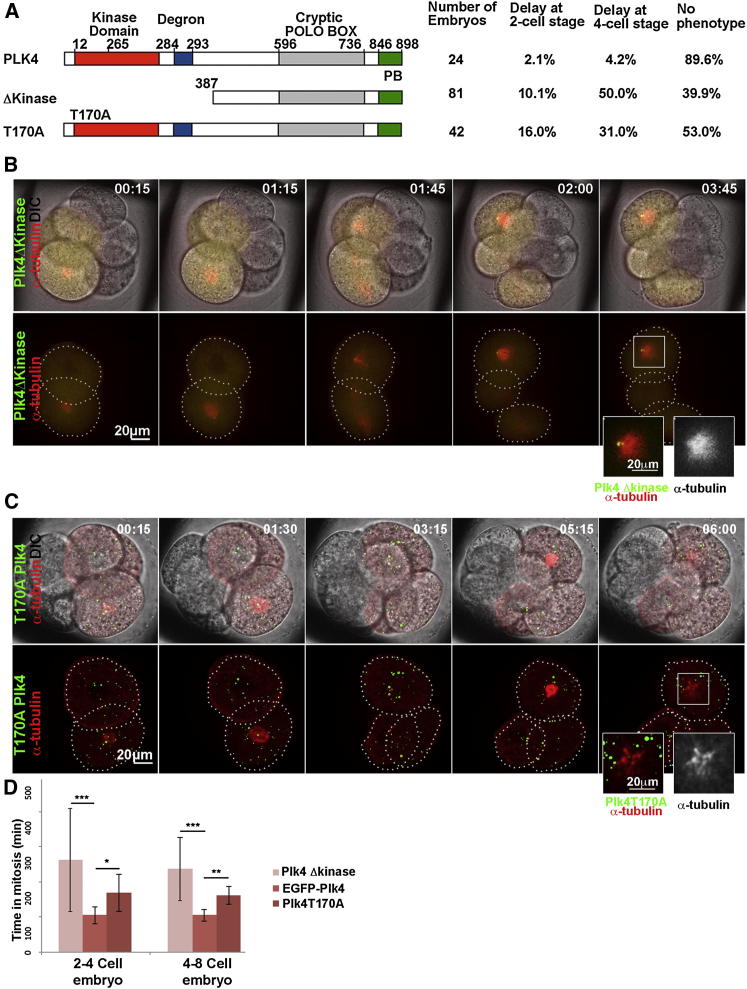
Assembly of Bipolar Spindle Depends on Plk4 Kinase Activity (A) Schematic showing domains of wild-type Plk4, Plk4ΔKinase, and T170A Plk4 point mutant. Distribution of phenotypes is indicated as a percentage. (B) Time-lapse series of an embryo with one two-cell blastomere injected with mRNA for ΔKinase (green) and α-tubulin-mcherry (red / monochrome in inset). Monoasters (inset) were consistently observed (44%). Time hr:min. (C) Time-lapse series of an embryo with one two-cell blastomere injected with mRNA for inactive Plk4, Plk4T170A (green), and α-tubulin-mcherry (red). A monoaster is visible in one of the two blastomeres. See also Movie S4. (D) Quantification of duration of mitosis from NEBD until cytokinesis. Expression of both, Plk4ΔKinase and Plk4T170A significantly prolongs the time spent in mitosis compared to EGFP-Plk4 control (^∗∗∗^p < 0.001, ^∗∗^p < 0.01, ^∗^p < 0.05).). Error bars indicate SD of average. See also Figure S3 and Movies S4 and S5.

These results indicate that Plk4 has an essential role in spindle formation through an effect on MT nucleation, but do not address whether this requires Plk4’s catalytic activity. To address this, we examined the consequences of expressing inactive mutant forms of Plk4 upon mitotic progression. Although endogenous Plk4 remains present, we anticipated the mutant constructs would act in a dominant negative manner because Plk4 has been shown to homodimerize (Guderian et al., 2010). We first found that injection of single 2-cell blastomeres with mRNA encoding Plk4Δkinase, a construct deleted for the entire kinase and degron domains of Plk4 led to a high proportion (60%, n = 49) of cells delayed in mitosis with monoastral spindles at the two- or four-cell stages, (Figure 4A, n = 81), resembling the Plk4 RNAi phenotype (Figures 4A, 4B, and 4D; Movie S4; Figures S3A and S3B). The injection of mRNA for Plk4T170A, in which the threonine of the activating T-loop is mutated to an alanine, led to an intermediate phenotype (47% showing abnormal mitosis; n = 42; Figures 4A, 4C, and 4D; Movie S4). Thus, catalytically inactive Plk4 interferes with the function of the endogenous wild-type protein.

The observation that spindle assembly is impaired by expression of these kinase-defective forms of Plk4 contrasts with the effects of similar mutants in cultured cells where kinase-dead Plk4 promotes centriole overduplication (Guderian et al., 2010). This is believed to reflect disruption of Plk4 self-phosphorylation, protecting Plk4 from SCF (Skp, Cullin, F-box containing)-mediated degradation (Guderian et al., 2010; Holland et al., 2012; Sillibourne et al., 2010). Thus, the loss of function phenotype seen in mouse embryo cells suggests that the regulation of Plk4 stability might not play an equally prominent role in spindle assembly as it does in centriole assembly in cultured cells (Cunha-Ferreira et al., 2009; Guderian et al., 2010; Holland et al., 2012; Rogers et al., 2009).

This interpretation is consistent with the finding that overexpression of a putative constitutively active form of Plk4, T170D, with the T-loop threonine mutated to the phosphomimic aspartic acid, had little effect on the mitotic progression (n = 26; Figures S3C and S3C′; Movie S5). Similarly, expression of a form with a mutated degron, which should be protected from SCF/proteasome-mediated degradation, also had little if any consequences on the MT nucleating activities of Plk4 (n = 27; Figures S3D and S3D′; Movie S5). Thus although Plk4 is required to assemble the bipolar mitotic spindle, excessive Plk4 has no negative consequence for the progression of blastomeres through their division cycles.

Together, the spindle defects observed following expression of Plk4Δkinase, as well as Plk4T170A, indicate that Plk4 relies on its kinase activity to regulate spindle assembly. This conclusion is further substantiated by studies of alternative mutants in the kinase domain (below). However, it is not possible to exclude an additional scaffolding role of the kinase and degron domains of Plk4, whose absence would cause the Δkinase Plk4 to exhibit a more extensive dominant mitotic phenotype than Plk4T170A.

### Partner of Plk4, Cep152, Is Required for Spindle Assembly

**Figure 5:**
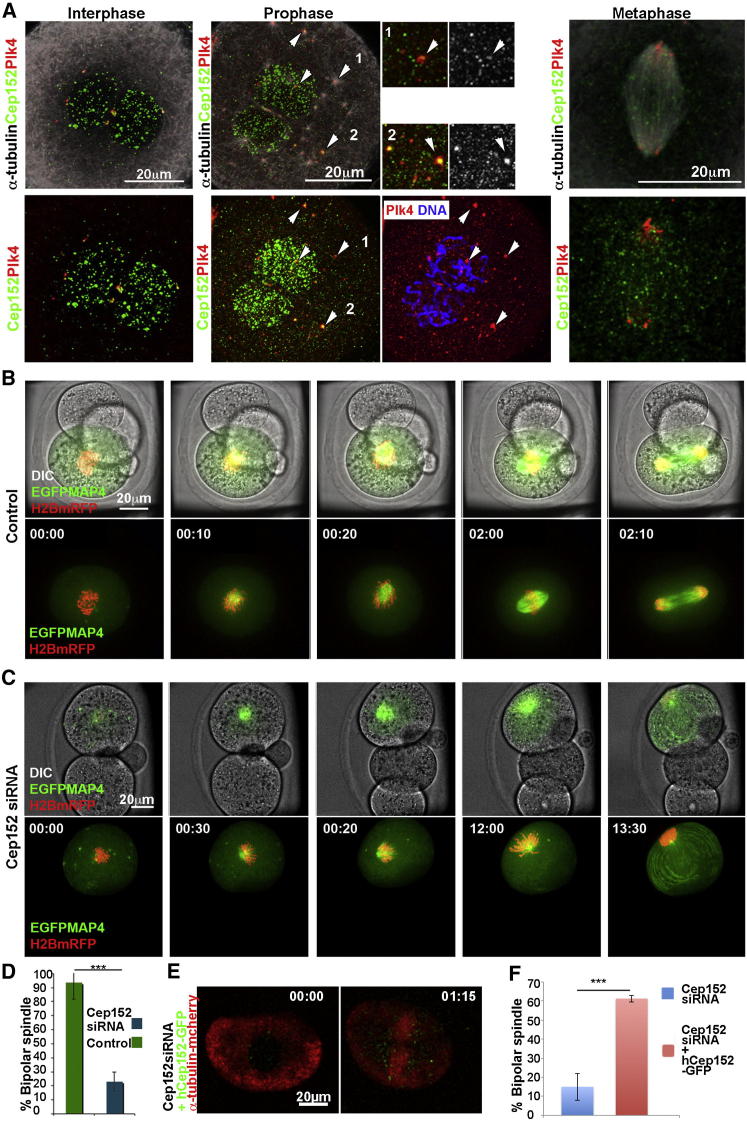
Downregulation of Cep152 Causes Monoaster Formation (A) Mouse zygotes stained to depict α-tubulin (white), Plk4 (red), and Cep152 (green). Cep152 predominantly localizes to the nucleus during interphase with some colocalization with Plk4 between the two pronuclei. Association of Cep152 with Plk4 at the MTOCs becomes widespread in prophase with MT nucleation (white arrows). Later in mitosis, Cep152 is preferentially in spindle MT-associated puncta. (B) Control two-cell stage embryo with one cell expressing histone H2B-mRFP (red) and EGFP-MAP4 (green). Time (hr:min) in relation to NEBD, 00:00. (C) Cep152 siRNA injected 2-cell blastomere labeled as in (B). MT nucleation is reduced and a single aster eventually forms. (D) Significant reduction in percentage of bipolar spindles formed after Cep152 RNAi compared to control (^∗∗∗^p < 0.001). Error bars represent SD of average. (E) Rescue of Cep152 phenotype (shown at two-cell stage) by coinjection of Cep152 siRNA with hCep152-GFP (green). MTs revealed by α-tubulin-mcherry (red). (F) Significant differences in percentage of bipolar spindles assembled following Cep152 depletion (n = 21) and rescue (n = 45; ^∗∗∗^p < 0.001). Error bars represent SD of average. See also Figure S4 and Movie S6.

Because Plk4 requires Cep152 for its centriolar functions (Cizmecioglu et al., 2010; Dzhindzhev et al., 2010; Hatch et al., 2010), we wished to determine whether Cep152 is also needed for Plk4-mediated spindle assembly without the involvement of centrioles. To address this, we first examined the spatial and temporal expression pattern of Cep152. We found that Cep152 first becomes enriched in the male and female pronuclei (n = 12) apparently due to the nuclear localizing properties of its N-terminal part (Figures S4A and S4B). It is only when zygotes start to enter prophase that Cep152 begins to localize with Plk4 at the cytoplasmic MTOCs (n = 6; arrowheads and insets, Figure 5A). Plk4 and Cep152 colocalize only upon NEBD, when MTOCs move into the vicinity of condensing chromosomes. This colocalization is also seen on MTOCs that are not at the vicinity of chromosomes at this stage. However, once the bipolar spindle becomes established, the association of Plk4 and Cep152 diminishes (n = 14): Plk4 clusters at the spindle poles and the majority of Cep152 associates with the spindle MTs (Figure 5A).

To determine whether colocalization of Cep152 and Plk4 at MTOCs might indicate shared function in spindle assembly, we depleted Cep152 in a single blastomere of two-cell embryos by injecting siRNA against Cep152 (n = 26). Both Cep152-depleted and control (n = 15) embryos were injected with mRNAs for EGFP-Map4 and Histone H2B-mRFP to mark MTs and chromosomes. We found that Cep152 RNAi eliminated Cep152 from the nucleus (Figures S4C and S4D) and led to diminished MT density in the vicinity of chromosomes, formation of monoastral spindles and, after a mitotic delay, severe cytokinesis defects (80%; n = 45; Figures 5B–5D; Movie S6), a phenotype very similar to that of Plk4 depletion. These defects could be rescued by coinjecting mRNA for human Cep152 tagged with GFP together with mouse siCep152 RNA (60%, n = 45; Figures 5E and 5F), demonstrating the specificity of the phenotype.

**Figure 6:**
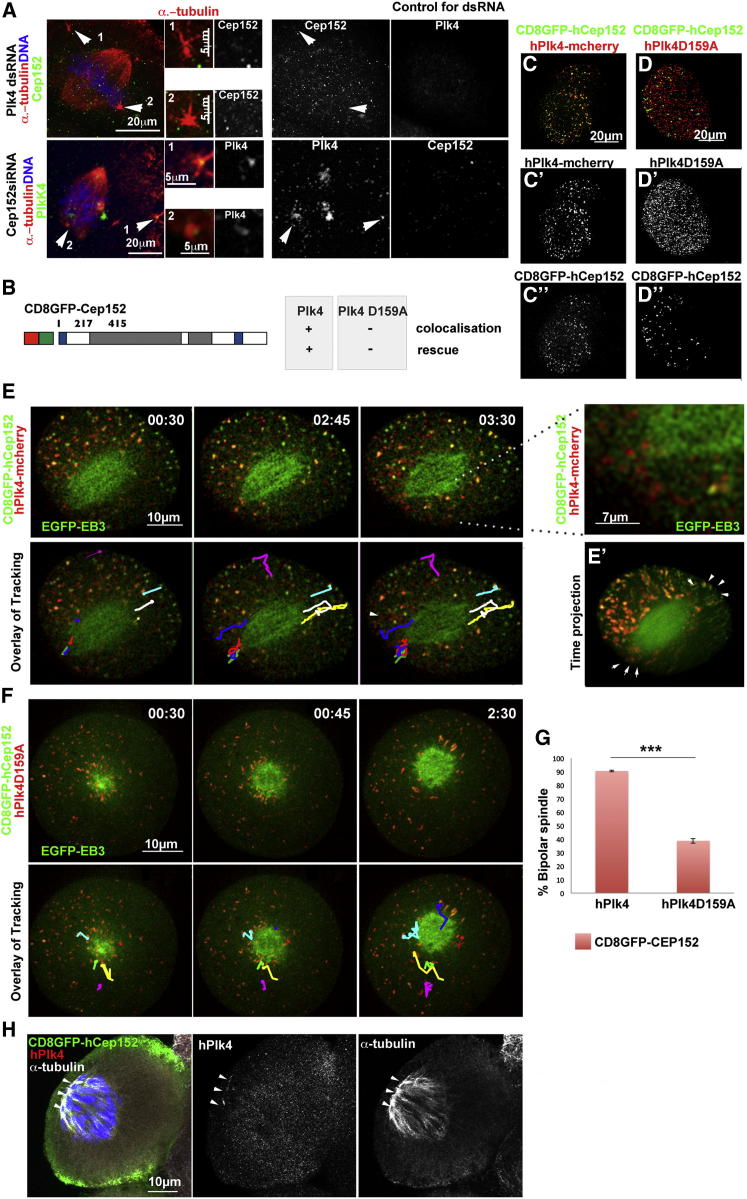
Interaction of Plk4 and Cep152 Is Required for Bipolar Spindle Formation (A) A single two-cell stage blastomere injected with Plk4 dsRNA (top panel) or Cep152siRNA (bottom panel) and stained for α-tubulin (red), Cep152 (green / monochrome), and Plk4 (green / monochrome). Note Plk4- or Cep152-depleted cells with monoastral spindles could not be examined because of their very small arrays of MTs from the MTOCs. Cep152 can be detected over the spindle after Plk4 depletion but not at the MTOCs (top panel, arrows). Plk4 is at MTOCs and spindle poles after Cep152 depletion (bottom panel, arrows). (B) Schematic showing membrane targeted hCep152 construct tagged with GFP and CD8 demonstrating ability to colocalize with human Plk4, human kinase dead Plk4 (D159A) and to promote bipolar spindle formation (rescue). (C) Four-cell stage embryo with one blastomere injected at the two-cell stage with CD8GFP-hCep152 (green/monochrome), and hPlk4-mcherry (red/monochrome), together with siRNA for Cep152 and Plk4 dsRNA. Colocalization of hCep152 with hPlk4 consistently observed. (D) Four-cell stage embryo with one blastomere injected at the two-cell stage with CD8GFP-hCep152 (green/monochrome), and kinase dead hPlk4D159A-mcherry (red/monochrome), together with siRNA for Cep152 and Plk4 dsRNA. Colocalization between the two proteins is no longer visible. (E) Two-cell stage embryo with one blastomere injected with CD8GFP-hCep152 (green), and hPlk4-mcherry (red), together with siRNA for Cep152 and Plk4 dsRNA. Coexpression of these proteins reverts the phenotype of Cep152 or Plk4 depletion; bipolar spindles (EGFP-EB3, green) form in proximity of the membrane, and blastomeres divide. Tracking of individual green/red dots (colored lines in bottom panel) shows their movement between the poles and membrane (magnification on right). See also Movie S7. (E′) Time projection (xyt) of average pixel intensity shows MT tracking close to membrane (arrows). (F) Two-cell stage embryo with one blastomere injected with CD8GFP-Cep152 (green), and kinase-dead hPlk4D159A-mcherry (red), together with siRNA for Cep152 and Plk4 dsRNA. Unlike wild-type, hPlk4D159A does not colocalize with Cep152, bipolar spindles are not assembled, and MT nucleation around chromosomes is observed. This indicates Plk4 activity is needed for functional interaction with Cep152. Tracking Plk4 shows random movements around the sites of MT nucleation (colored lines in bottom panel). See also Movie S7. (G) Quantification of the phenotype rescue by expression of CD8GFP-hCep152 and hPLK4-mcherry (n = 12) or CD8GFP-hCep152 and kinase-dead hPlk4D159A (n = 10). Rescue is significant (^∗∗∗^p < 0.001; bars indicate SD of average. (H) Early mouse embryo depleted for Cep152 and Plk4 in one two-cell embryo blastomere by injection of Plk4 dsRNA and Cep152siRNA. Blastomere was coinjected with CD8-hCep152 and humanPlk4. Embryos were stained to depict CD8-GFP-hCep152 (green/monochrome), α-tubulin (white), Plk4 (red/monochrome), and DNA (blue). Plk4 associates with CD8-hCep152 at the membrane in interphase and spindles are formed with one pole clearly at the membrane (white arrowheads) and the other pole unfocused. Chromosomes are positioned closer to this pole.

The similarity of the Cep152 and Plk4 depletion phenotypes and the colocalization, of the two proteins upon NEBD suggested they might work in partnership to mediate spindle assembly. To explore the interdependencies of Plk4 and Cep152 localization, we depleted either Plk4 or Cep152, and then examined the localization of the other. We found that in Cep152 depleted cells, Plk4 still localized to the MTOCs (n = 15), indicating that presence of Plk4 by itself is insufficient to drive spindle formation and that its function is dependent on Cep152 (Figure 6A). In contrast, Cep152 was no longer observed at the MTOCs following Plk4 depletion (n = 8), although it was still found in interphase nuclei and over the mitotic spindle, indicating that Cep152 requires Plk4 to localize to the MTOCs. This suggests that in mouse embryo, Plk4 is maintained at the MTOCs by a protein other than Cep152 and once Plk4 becomes localized to MTOCs and the nuclear envelope has broken down, Cep152 is able to interact with Plk4 to support MTOC function in spindle assembly.

### Plk4 and Cep152 Interaction Requires Plk4 Kinase Activity and Is Necessary for Spindle Assembly

The above results suggest that interactions between Cep152 and Plk4 enable MTs to be established between MTOCs and chromosomes at early stages of mitotic progression. To test this hypothesis and to further understand whether MT nucleation at the MTOCs specifically requires the Cep152-Plk4 interaction, we wished to target Cep152 to ectopic sites and determine its ability to interact with Plk4. To this end, we fused an integral membrane component, the mouse T cell receptor CD8, with the full coding region of human Cep152 (hCep152). In addition, we inserted a GFP tag between the two genes to follow the hybrid protein in live embryos (Figure 6B). We injected mRNA for CD8-GFP-tagged hCep152, together with mRNA for mcherry-tagged humanPlk4 (hPlk4), into one two-cell blastomere that we earlier depleted for endogenous Cep152 and Plk4, by RNAi (n = 12). We found that the CD8-tagged Cep152 localized to both the plasma membranes and numerous putative membranous sites throughout the cytoplasm and that hPlk4 was colocalized at all of these sites (Figure 6C), confirming the interaction between Cep152 and Plk4. Remarkably, coexpression of CD8-tagged hCep152 and hPLK4 was able to rescue the formation of bipolar spindles. Typically these spindles had one of their poles closer to the membrane with the other pole being unfocused. Immunostaining confirmed that MTs were nucleated from the ectopic CD8-hCep152 and hPlk4 aggregates at entry into mitosis. Notably the chromosomes were skewed toward the ectopic MT nucleation sites (100%; n = 10; Figure 6H). Of all the hPlk4 particles, 97.1% ± 2.9% colocalized with the membrane-targeted Cep152 (n = 12). Tracing the positions of those CD8-hCep152::hPlk4 particles in the vicinity of the spindle poles revealed their highly dynamic movements between the poles and the plasma membrane over distances of up to 5 μm (Figure 6E; Movie S7). Immunostaining experiments confirmed that MTs were nucleated from the ectopic CD8-hCep152 and hPlk4 colocalizing dots at entry into mitosis (Figure 6H). These results indicate that the ectopic CD8-hCep152 and hPlk4 interaction is sufficient to promote spindle assembly.

To confirm the specificity of the above result and determine whether activity of Plk4 is required, we coexpressed CD8-tagged hCep152 with a kinase-dead form of hPlk4 (hPlk4D159A). In this case only 0.9% ± 0.5% of kinase-dead hPlk4 particles colocalized with CD8-tagged hCep152 at cytoplasmic membranes and instead, Plk4 was diffused within the interphase cytoplasm and clustered around the periphery of the mitotic chromosomes and MTs (n = 10; Figure 6F; Movie S7). These kinase-dead hPlk4 particles were also considerably less dynamic than the spindle pole-associated CD8-hCep152::hPlk4 particles in control cells. Notably, the monoastral phenotype of the double depletion was not rescued when the kinase-dead form of Plk4 was expressed in this way (Figure 6G). These results indicate that Plk4 activity is required both to establish colocalization with Cep152 and for bipolar spindle formation.

## Discussion

**Figure 7:**
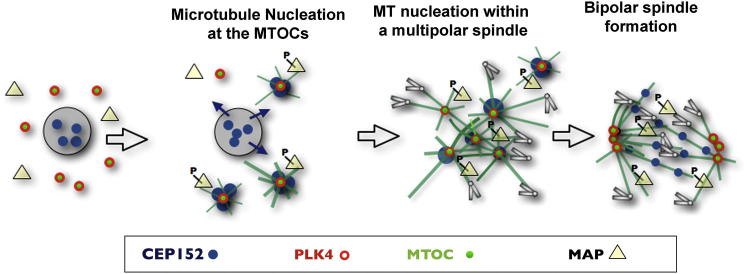
Schematic Representation of Early Events in Acentriolar Spindle Assembly in Mouse Plk4 localizes at MTOCs from interphase, in contrast to Cep152, which is maintained in the nucleus. Once Cep152 is released from the nucleus, it interacts with Plk4 localized at MTOCs at the time of nuclear envelope breakdown. We propose that Cep152 provides a platform for Plk4 to either interact with its substrates, and/or to be activated itself, gaining the ability to trigger MT nucleation. MT-associated proteins will be activated during this process, either as direct or indirect consequence of Plk4 phosphorylation. We do not exclude the possibility of other proteins as substrates, such as spindle assembly factors, known to be active specifically at the entry into mitosis. After bipolar spindle assembly, Plk4 remains at the spindle poles, while Cep152 is no longer needed at these sites and is displaced over the spindle.

We show that Plk4 kinase, primarily recognized thus far for its role in de novo centriole formation, is also essential for spindle assembly in the absence of centrioles (Figure 7). Our results indicate that maternally provided Plk4 is carried by cytoplasmic MTOCs that, following NEBD, are able to recruit Plk4 partner protein, Cep152. A subset of these MTOCs then form the foci of an increased mass of MTs whose plus ends lie in the vicinity of chromosomes. Plk4 is essential to ensure sufficient density and dynamicity of these MTs around the condensing chromosomes. In the absence of Plk4, the nucleated MTs cannot grow to become organized into bipolar arrays to promote spindle formation and, instead, mono-astral structures form. After a prolonged delay in mitosis, the Plk4-depleted cells attempt monopolar cytokinesis that is detrimental to further development.

The spindle assembly functions we now ascribe to Plk4 are mediated through its association with Cep152. Thus the Plk4-Cep152 partnership has essential functions beyond centriole duplication in governing the nucleation and organization of MTs in mitosis. It has previously been proposed that Plk4 kinase itself is required for the anaphase-promoting complex-dependent destruction of cyclin B1 and exit from mitosis in the postimplantation mouse embryo (Hudson et al., 2001). Moreover, it was also reported that Plk4 phosphorylates the transcription factor, Hand1, to mediate its release from the nucleolus and promote trophectoderm differentiation (Martindill et al., 2007). Thus, it appears that Plk4 might play multiple functions in both cellular as well as developmental processes.

The role of Plk4 in driving centriole biogenesis is well established (Bettencourt-Dias et al., 2005; Cunha-Ferreira et al., 2009; Eckerdt et al., 2011; Guderian et al., 2010; Holland et al., 2010; Rodrigues-Martins et al., 2007; Rogers et al., 2009). However, our data indicate that its presence is not enough for centriole formation in the early mouse embryo. This could be in part because its partner, Cep152, is predominantly within the interphase nucleus and only transiently associates with Plk4 at NEBD but then localizes to the spindle MTs. Moreover, we find that other centriolar proteins required for procentriole formation appear to be rate-limiting until the 32- to 64-cell stage (Figure S1J). This contrasts with the eggs of *Drosophila* and *Xenopus* that rely heavily upon maternal contribution of proteins required for very rapid cell cycles perhaps explaining why overexpression of Plk4 can drive centriole formation in these systems (Rodrigues-Martins et al., 2007; Eckerdt et al., 2011).

The precise mechanism by which Plk4 promotes centriole formation is still not clear. Its substrates include some centrosomal proteins, such as Sas6 in *C. elegans*, and the γ-Turc component, GCP6, in mammalian cells but the immediate consequences of their phosphorylation are not known (Bahtz et al., 2012; Hatch et al., 2010; Nigg and Stearns, 2011). Similarly, we can only hypothesize about the functional consequences of Plk4-mediated protein phosphorylation in spindle formation. It is possible that defective MT nucleation and dynamics, which are the earliest effects of Plk4 depletion or kinase inactivation, are manifestations of the same primary defect, namely retarded MT growth at the plus tips. These could be mediated by Plk4 either directly or indirectly regulating the behavior of MT-associated proteins (MAPs). Indeed Plk4’s partner Cep152 could be a candidate intermediate molecule because its *Drosophila* counterpart, Asl, has been implicated in regulating MT nucleation and in forming a complex with the MT-binding Sas4 (Bonaccorsi et al., 1998; Varmark et al., 2007; Dzhindzhev et al., 2010).

The later consequences of Plk4 depletion in mitosis could be secondary to the failure of MT growth or they could reflect additional roles for Plk4. In the absence of dominant polar MTOCs, the establishment of spindle bipolarity depends upon the ability of MTs to organize themselves into antiparallel arrays, a process facilitated by the Eg5 kinesin-like protein (Walczak et al., 1998). Without Plk4, the mitotic MTs of early mouse embryo cells remain in monoastral structures. While this could be a consequence of the reduced numbers and dynamicity of MTs, it is also possible that Plk4 may be required to control motor proteins that regulate bipolarity (Walczak et al., 1998). The lack of tension on chromosomes in such monopolar arrays would account for the ensuing prolonged delay in mitosis by failure to satisfy the spindle assembly checkpoint. When Plk4-depleted cells eventually slip through the checkpoint, they attempt monopolar cytokinesis. This could reflect misregulation of Ect2, a Rho GEF that controls the contractile ring assembly at the equatorial cortex, which has been reported to be a Plk4 substrate (Holland et al., 2012; Rosario et al., 2010). However, a direct role for Plk4 in cytokinesis remains uncertain and we note that cytokinesis defects similar to those observed here have been reported as a secondary consequence of spindle monopolarity in cultured cells (Hu et al., 2008).

Nucleation of MTs in the vicinity of chromosomes appears to coexist alongside centrosome-driven spindle assembly in most cell types (Meunier and Vernos, 2012). It can be directly followed if MTs are depolymerized and allowed to regrow. Under these conditions, intense MT asters form around centrosomes and additional smaller asters appear close to the chromatin (Meunier and Vernos, 2011). These asters appear to be the functional counterpart of the MTOCs of the mouse embryo and Plk4 might play a role in regulating their function, a possibility supported by the finding that Plk4 depletion leads to monoaster formation (Bettencourt-Dias et al., 2005; Habedanck et al., 2005). Although this was attributed to the loss of centrioles, we find that when centrioles are eliminated after depletion of Sas6 in U2OS cells, monoastral spindles are not assembled (not shown). Together these results open a possibility that Plk4 might also function in chromosome-mediated MT assembly in cells that have centrosomes organized around centrioles.

A mitotic role of Plk4, in addition to its G1/S function in centriole duplication, would be consistent with its reported activity profile. A phosphomodified activated form of Plk4 has been shown to be restricted to the mother centriole as centrioles duplicate in G1/S and to the daughter centriole in G2, but its activity only rises to its maximum in mitosis (Sillibourne et al., 2010). The restriction of Cep152 to the nucleus in early mouse embryo provides the means of regulating formation of a functional complex of Plk4 at MTOCs upon NEBD. However, the experimental tethering Cep152 to cytoplasmic membranes indicates that Plk4 can be recruited to these ectopic sites in interphase. Because MT arrays are only observed at these ectopic sites at the entry into mitosis, there must be additional mechanisms that regulate the ability of the Plk4-Cep152 complex activated at mitosis (Figure 7).

In conclusion, the results we present here identify a contributory mechanism for regulating spindle formation where no distinct centrosomes define the spindle poles, as is the case in early mouse development. Spindle formation in these cells first requires the activation of MTOCs in the vicinity of the mitotic chromosomes in a process that requires Plk4 to form an active complex with Cep152. The resulting MTs associate with chromosomes at their plus ends and the MTOCs at their minus ends. Once this complex has mediated the nucleation of sufficient MTs to adopt antiparallel arrays, it dissociates and Cep152 is displaced over the spindle. It will be of future interest to determine how these Plk4-Cep152 mediated events relate to those regulated by RanGTP, also essential for spindle formation in these cells (Dumont et al., 2007; Schuh and Ellenberg, 2007) and to identify other partner proteins and mitotic substrates of Plk4 in the spindle assembly process. Although spindle assembly takes place in the mouse embryo through the chromosome-mediated pathway, our findings demonstrate that it requires the participation of MTOC associated Plk4, even in the absence of centrioles.

## Experimental Procedures

### Embryo Collection and Culture

C57Bl6xCBA mice were mated following superovulation of females and embryos were collected in M2 media, following standard procedures as previously published (Bischoff et al., 2008; Piotrowska-Nitsche and Zernicka-Goetz, 2005). Isolated embryos were cultured in KSOM media under paraffin oil at 37°C in a 5% CO_2_ atmosphere. All experimentation with mice was carried out following requirements of the UK Home Office under a Project License held by M.Z.-G.

### Microinjections

For microinjection of mRNA, in vitro transcription of Sfil-linearized RN3P plasmids was performed using mMessage mMachine T3 Polymerase (Life Technologies) according to the manufacturer’s instructions. Microinjection of in vitro transcribed mRNAs, or Cep152 siRNAs (QIAGEN; for sequences of siRNAs, see below) were performed as previously established (Zernicka-Goetz et al., 1997). To monitor cell division from the two-cell stage and onward, a single random blastomere was microinjected with the RNAs.

### Live Embryo Imaging

Time-lapse images were collected every 10–15 min for the green and red channels on an inverted Zeiss Axiovert with a spinning disk confocal head (Intelligent Imaging Solutions) using a 63×/1.3 water objective. Each Z stack comprises twenty images at 3 μm intervals. Image processing and analysis was performed using Slidebook 5.0.0.20. Data stacks were deconvolved with Huygens Professional (Scientific Volume Imaging). Image sequence analysis, MT Kymograph, and video assembly were performed with ImageJ Software (NIH).

### Antibodies

DNA encoding a polypeptide corresponding to the internal 466 amino-acids (residues 313–778) of mouse PLK4 was cloned into the expression vector pDEST17 (Invitrogen) for expression of a Histidine terminally tagged peptide in *Escherichia coli.* The bacterially expressed polypeptide was used to raise the rat antiserum, anti-Plk4. Primers used for subcloning the DNA encoding the peptide were mPLK4_For and mPLK4_Rev (Table S1).

We used the following antibodies: rat antimouse PLK4 (see above) 1:2,000; rat anti-α-tubulin-YL1/2 (Oxford Bioscences,1:50); mouse anti-α-tubulin-DM1A (Sigma, 1:10,000); mouse anti-γ-tubulin-GTU88 (Sigma,1:25); rabbit anti-γ-tubulin T3559 (Sigma,1:500); mouse anti-centrin2 S3332(Santa Cruz Biotechnology, 1:500); rabbit anti-CP110 (1:500); mouse anti-Sas6 (1:200, Abnova); and anti-humanCep152 (Cizmecioglu et al., 2010) against C- or N-terminal regions (a kind gift from I. Hoffmann). The secondary antibodies used (1:2,000 for immunofluorescence) were conjugated with Alexa 488, Alexa 568, or Alexa 647 (Invitrogen) and had minimal cross-reactivity to other species.

### RNA Constructs

Primers used for Plk4 dsRNA production: T7 PLK4_300 and SP6 PLK4_870 (Table S1). DsRNAs were transcribed using RiboMAX Large-scale RNA Production Systems SP6 and T7 (Promega), following manufacturer’s instructions. Cep152 Knockdown by siRNA was performed with FlexiTube GeneSolution GS99100 (QIAGEN).

### cDNA Constructs

Human Plk4 (NM_014264, IMAGE: 5273226) and mouse Plk4(BC051483, IMAGE:1379362) were subcloned into RN3P for in vitro transcription of mRNA. Human Cep152 (BC117182, IMAGE: 40125733) was subcloned in the Gateway system and the CD8GFP-humanCep152 construct was generated using a pMT vector with a CD8::EGFP-Gateway cassette (D’Avino et al., 2006). The coding region of CD8GFP-humanCep152 was PCR amplified and cloned into RN3P vector for in vitro transcription. All the constructs were sequenced on both strands.

### Immunofluorescence

Embryos were fixed in ice-cold methanol: DMSO (9:1) for 30 min, followed by permeabilization in 1× PBS 0.1% BSA, 10% fetal bovine serum (FBS), and 0.5% Triton X-100 for 1 hr and blocking in 1× PBS, 0.1% BSA, 0.1% Tween, and 10% FBS for another 1 hr. Incubation in primary and secondary antibodies was carried out in 1× PBS, 0.1% BSA, 0.1% Tween, and 10% FBS. Washes were performed using 1× PBS, 0.1% BSA, and 0.1% Tween. Embryos were mounted in Vectashield Mounting Medium with DAPI (Vector Laboratories). Images were collected on a Zeiss LSM 510 Meta Laser Scanning Confocal Microscope using 63×/1.4 or 100×/1.4 oil objectives, and the LSM 510 Version 4.2 software. Images were deconvolved using Huygens Professional software; processing and analysis was performed with ImageJ Version 1.45 s and Adobe Photoshop CS5. All images shown are the projections of optical sections.

### Structured Illumination Microscopy and Data Processing

Images were acquired using DeltaVision OMX3D-SIM System V3 (Applied Precision). All data were captured using an Olympus 100× 1.4 NA oil objective, 488 nm/593 nm/405 nm laser illumination and standard excitation and emission filter sets as described previously (Fu and Glover, 2012).

### Statistical Analysis

Statistical analysis was carried out using the Student’s t test with a two-tailed distribution. All data points were included in statistical analyses and the experiments were repeated at least three times independently. In the graphs, the average is shown and error bars correspond to SD of the average.

### Site-Directed Mutagenesis

For mutagenesis, the QuikChange II XL Site-Directed Mutagenesis Kit (Stratagene) was used according to the manufacturer’s instructions. Primers used were as follows: T170AFOR and T170AREV to produce the Plk4T170A mutant form; T170DFOR and T170DREV to generate the Plk4 T170D variant; DegronFOR and DegronREV to generate the Plk4 nondegradable form; and D159AFor and D159Rev to generate the kinase-dead human Plk4. The sequence of each of the oligonucleotides referred to above is shown in Table S1.

### Electron Microscopy

Embryos were transferred to 2.5% glutaraldehyde in PBS for 30 min. Embryos were kept in 1× PBS, 03% Tween-20, and 2.5% glutaraldehyde, and stained for 1 hr in osmium tetroxide before embedding in Epon-Araldite prior to sectioning.

## Supplemental Information

Document S1. Figures S1–S4 and Table S1

Movie S1. Plk4 Associates with Acentriolar MTOCs of the Early Mouse Embryo, Related to Figure 1 Time-lapse imaging of zygotes expressing α-tubulin-mcherry (red) and Plk4-GFP (green). Z stacks are composed of 20 optical sections covering 60 μm and were acquired every 15 min. NEBD corresponds to time 00:00 (hr:min).

Movie S2. Plk4 Depletion Results in Monopolar Spindle Formation in the Early Mouse Embryo, Related to Figure 2 Time-lapse imaging of 2- to 4-cell stage transition. Control: One of the blastomeres was injected with mRNA encoding EGFP-MAP4 (green) and histone H2B-mRFP (red) to follow the dynamic of MT nucleation and spindle assembly at the same time as chromosome condensation and sister chromatid segregation. Below are the merged channels for EGFP-MAP4 and histone H2B-mRFP, and above are the same channels merged with DIC (black and white). Plk4 RNAi example 1: For Plk4 depletion, the blastomere was coinjected with Plk4 dsRNA, together with mRNA encoding EGFP-MAP4 (green) and histone H2B-mRFP (red). MT nucleation activity from MTOCs is observed at NEBD, but no bipolar spindle is formed. The blastomere eventually exits M phase without undergoing cytokinesis. Below are the merged channels for EGFP-MAP4 and histone H2B-mRFP, and on the top are the same channels merged with DIC (black and white). Plk4 RNAi example 2: In this example for Plk4 depletion, MT nucleation is observed over the chromosomes without the formation of a bipolar spindle. The blastomere exits M phase without undergoing normal cytokinesis. Z stacks are composed of 20 optical sections 3 μm apart. Images were acquired every 10 min. NEBD corresponds to time 00:00 (hr:min). Time-lapse imaging of 4- to 8-cell stage transition. Control: One of the blastomeres was injected with mRNA encoding α-tubulin-mcherry to follow the dynamics of spindle assembly (below). Above are DIC images at the corresponding time points. The embryos were imaged during the 4- to 8-cell stage transition. Plk4 RNAi: This movie is followed by another time-lapse imaging of a 4-cell embryo in which one of the blastomeres was depleted for Plk4 by injection of Plk4 dsRNA at the 2-cell stage. The embryo was coinjected with mRNA encoding α-tubulin-mcherry to follow the dynamics of spindle assembly (bottom). DIC images of the embryo collected at the corresponding time points are shown on top. The embryos were imaged from the 4- to the 8-cell stage. See also Figure S2. Time-lapse imaging of 8- to 16-cell stage transition. Plk4 RNAi: One of the blastomeres was depleted for Plk4 by injection of Plk4 dsRNA at the 2-cell stage. The embryo was coinjected with mRNA encoding α-tubulin-mcherry to follow the dynamics of spindle assembly (left). DIC images of the embryo collected at the corresponding time points are shown on top. Human Plk4-GFP rescue: Time-lapse imaging of an 8-cell embryo in which one of the blastomeres was depleted for Plk4 by injection of Plk4 dsRNA at 2-cell stage. The embryo was coinjected with mRNA encoding α-tubulin-mcherry and humanPlk4-GFP to determine whether the human Plk4 could rescue the Plk4 dsRNAi depletion. Below are the α-tubulin-mcherry channel (red) and humanPlk4-GFP (green). Above are DIC images of the embryo merged with α-tubulin-mcherry and humanPlk4-GFP channels. These previous embryos were imaged during the 8- to 16-cell transition. Z stacks are composed of 20 optical sections 3 μm apart. Images were acquired every 15 min. NEBD corresponds to time 00:00 (hr:min).

Movie S3. Plk4 Depletion Dramatically Reduces Kinetics of Microtubule Regrowth, Related to Figure 3 On the left side is time-lapse imaging of the 2- to 4-cell transition. One of the blastomeres was injected with mRNA codifying humanPlk4-mcherry (red) together with EGFP-EB3 (inverted black) at 2-cell stage. When the first MT nucleation at MTOCs was observed, embryos were subjected to cold treatment. MT regrowth occurs around PLK4 foci around chromosomes. On the right side is time-lapse imaging of a 4-cell embryo. At the 2-cell stage, one of the blastomeres was depleted for Plk4 by injection of Plk4 dsRNA. The blastomere was coinjected with mRNA codifying EGFP-EB3 (inverted black). After cold treatment, during regrowth assays, density of MTs was reduced. Z stacks are composed of four optical sections 1 μm apart. Images were acquired every 3 s. NEBD correspond to time 00:00 (min:s).

Movie S4. Expression of Kinase-Defective Plk4 Results in Monopolar Spindle Formation in the Early Mouse Embryo, Related to Figure 4 Time-lapse imaging of a 4-cell embryo in which one of the blastomeres was injected with mRNA encoding EGFP-ΔkinasePlk4 together with α-tubulin-mcherry at the 2-cell stage. Below are α-tubulin-mcherry (red) and EGFP-ΔkinasePlk4 (green). Above are DIC images of the embryo merged with α-tubulin-mcherry and EGFP-ΔkinasePlk4 channels. The lower cell completes mitosis and cell division; a monoastral spindle is formed in the upper cell. Time-lapse imaging of a 4-cell embryo in which one of the blastomeres was injected with mRNA codifying EGFP-T170APlk4 together with α-tubulin-mcherry at the 2-cell stage. Below are the α-tubulin-mcherry (red) and EGFP-T170APlk4 (green) channels. Above are DIC images of the embryo merged with α-tubulin-mcherry and EGFP-T170APlk4 channels. The embryos were imaged during the 4- to 8-cell transition. Z stacks are composed of 20 optical sections 3 μm apart, and images were acquired every 15 min. NEBD corresponds to time 00:00 (hr:min).

Movie S5. Constitutively Active or Degron Mutants of Plk4 Do Not Affect Spindle Formation in the Early Mouse Embryo, Related to Figure 4 Time-lapse imaging of a 4-cell embryo in which one of the blastomeres was coinjected with mRNA encoding a constitutively active form of PLK4 with a mutation at the T loop, T170DPlk4, and mRNA for α-tubulin-mcherry at the 2-cell stage. α-tubulin-mcherry (red) and EGFP-T170DPlk4 (green) channels are shown. This movie is followed by a time-lapse imaging of a 4-cell embryo in which one of the blastomeres was injected with mRNA encoding a nondegradable, degron-mutant form of PLK4, EGFP-DegronPlk4 together with α-tubulin-mcherry at the 2-cell stage. α-tubulin-mcherry (red) and EGFP-DegronPlk4 (green) channels are shown. The embryos were imaged during the 4- to 8-cell transition. Z stacks are composed of 20 optical sections 3 μm apart. Images were acquired every 15 min. NEBD corresponds to time 00:00 (hr:min).

Movie S6. Depletion of Cep152 Results in Monopolar Spindle Formation in the Early Mouse Embryo, Related to Figure 5 On the left is time-lapse imaging of a 2-cell embryo in which one of the blastomeres was injected with mRNA encoding EGFP-MAP4 (green) and histone H2B-mRFP (red) to follow the dynamic of MT nucleation and spindle assembly simultaneously with chromosome condensation and sister chromatid segregation. Below are the merged channels for EGFPMAP4 and histone H2B-mRFP. Above are the corresponding channels merged with DIC. On the right is time-lapse imaging of a 2-cell embryo in which one of the blastomeres was depleted for Cep152 by siRNA and coinjected with mRNAs encoding EGFP-MAP4 (green) and histone H2B-mRFP (red). Below are the merged channels for EGFP-MAP4 and histone H2B-mRFP. Above are the corresponding channels merged with DIC. The embryos were imaged at the 2- to 4-cell stage. Z stacks are composed of 20 optical sections 3 μm apart. Images were acquired every 10 min. NEBD corresponds to time 00:00 (hr:min).

Movie S7. Membrane-Targeted Cep152 Directs Active, but Not Inactive, Plk4 to This Ectopic Site to Rescue Double Depletion of Endogenous Plk4 and Cep152, Related to Figure 6 On the left is time-lapse imaging of a 2-cell embryo in which one of the blastomeres was depleted for Cep152 by siRNA and for PLK4 by dsRNAi. The embryo was coinjected with mRNA encoding CD8GFP-humanCep152 (green) and humanPLK4-mcherry (red) to determine whether these constructs could rescue Plk4 and Cep152 depletion. EGFP-EB3 mRNA (green) was also coinjected to visualize MT nucleation. Bipolar spindles are formed with their poles in proximity to the membrane, and blastomeres are able to divide. On the right is time-lapse imaging of a 2-cell embryo in which one of the blastomeres was depleted for Cep152 by siRNA and for PLK4 by dsRNAi. The embryo was coinjected with mRNA encoding CD8GFP-humanCep152 (green) and the kinase dead humanPLK4D159A-mcherry (red). MT nucleation was observed by expression of EGFPEB3 mRNA (green), which was coinjected. Although MT nucleation is observed, bipolar spindle does not assemble and cytokinesis fails. The embryos were imaged during the 2- to 4-cell stage. Z stacks are composed of 20 optical sections 3 μm apart. Images were acquired every 15 min. NEBD corresponds to time 00:00 (hr:min). This first set of movies is followed by substacks showing colocalizing CD8GFP-Cep152 (green) and humanPLK4 wild-type (red, on the left) or the kinase dead Plk4D159A (red, on the right) identified and tracked through mitosis. A movement between membrane and spindle poles is observed if wild-type human Plk4 is injected. Conversely, kinase-dead PLK4D159A localizes around the MT nucleation sites, undergoing random movement.
