# Supplementary material for: Spindle Formation in the Mouse Embryo Requires Plk4 in the Absence of Centrioles
Source: Dev Cell. 2013 Dec 9;27(5):586–97. doi: 10.1016/j.devcel.2013.09.029 (PMC3898710; doi:10.1016/j.devcel.2013.09.029)
Supplement: Document S1. Figures S1–S4 and Table S1 [file mmc1.pdf]

Developmental Cell, Volume *27*

Supplemental Information

Spindle Formation in the Mouse Embryo

Requires Plk4 in the Absence of Centrioles

Paula A. Coelho, Leah Bury, Bedra Sharif, Maria G. Riparbelli, Jingyan Fu, Giuliano Callaini, David M. Glover, and Magdalena Zernicka-Goetz

Inventory of Supplementary Information

Figures

- Figure S1, related to Figure 1
- Figure S2, related to Figure 2
- Figure S3, related to Figure 4
- Figure S4, relating to Figure 5

Movies

- Movie S1, related to Figure1
- Movie S2, related to Figure 2
- Movie S3, related to Figure 3
- Movie S4, related to Figure 4
- Movie S5, related to Figure 4
- Movie S6, related to Figure 5
- Movie S7, related to Figure 6

Table

- Table S1, related to Experimental Procedures

Supplementary Data

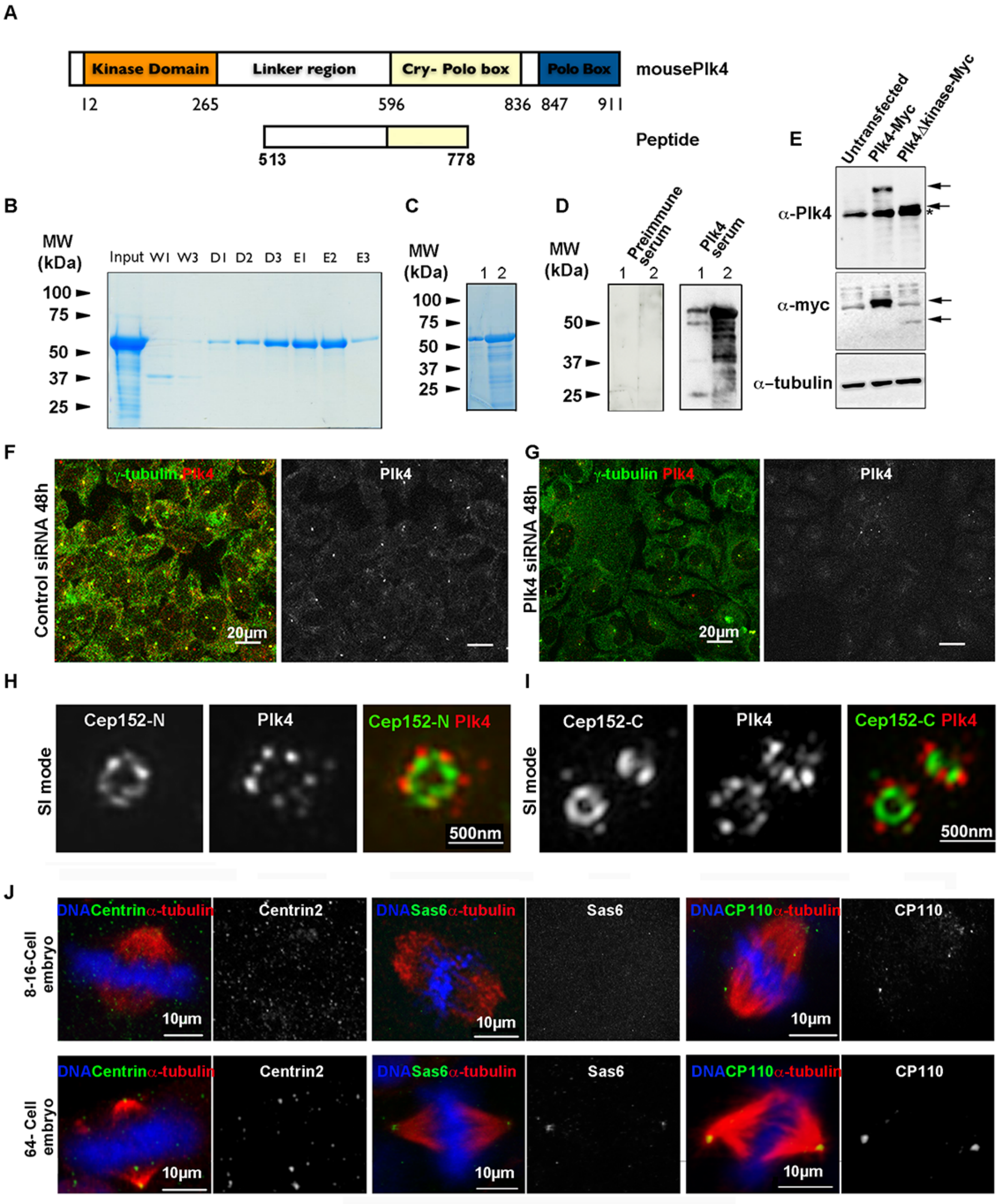

Figure S1, related to Figure1. Plk4 antibody recognizes endogenous Plk4 at the centrioles of mammalian cells. (**A**) The antibody generated to mouse Plk4 was raised against a peptide spanning aminoacids 513-778 and corresponding to parts of the linker region and cryptic polo box domains of Plk4. This region shares a high degree of homology with the human orthologue and a corresponding region was previously used to generate an antibody against human Plk4

(Habedanck et al., 2005).(**B**) Purification of Plk4(513-778aa)-His. Plk4(513-778aa)-His was expressed in *E.coli* BL21 and purified by Nickel-column affinity chromatography using increasing pH for elution (lanes D1-E3). Most protein was eluted with buffer E. **(C-D)** Purified Plk4 peptide separated by SDS-PAGE. 25µg (lane 1) and 50µg (lane 2) of protein were applied and visualized by Commassie staining (**C**), or Western Blotting (**D**). For Western Blotting, both pre- immune serum (left panel) and the fourth bleed of the immunized rat (right panel) are shown. The antibody recognizes the peptides used for immunization, whereas no band is detected using the corresponding pre-immune serum. (**E**) HEK 293 cells either untransfected, transfected with full length Plk4-Myc, or with Myc-tagged Plk4 lacking the kinase domain (Plk4Δkinase). Lysates were prepared and subjected to western blotting, using the indicated antibodies. The anti-Plk4 antibody specifically detects both exogenous Plk4 constructs. Arrows indicate full length Plk4 and Plk4-Δkinase. A non-specific band (asterisk) is visible also in the lysate of untransfected HEK 293 cells. (**F-G**) U2OS cells were either treated with control siRNA (**F**), or with Plk4 siRNA (**G**), and stained to reveal α-tubulin (green) and Plk4 (red and monochrome). Plk4 is extensively depleted from cells 48 h after transfection with Plk4 siRNA. The immunostaining of Plk4 at the centriole is lost after knockdown by siRNA but retained in control siRNA cells Plk4. (**H, I**) Localization of the endogenous Plk4 to the centriole of U2OS cells by three-dimensional-structured illumination microscopy (3D-SIM). (**H**) Staining with anti-Plk4 antibody (red); N-terminal region of Cep152 (green). The Plk4 partially colocalizes with Cep152. (**I**) Staining to reveal Plk4 (red) and the C-terminal region of Cep152 (green). The C-terminal domain of Cep152 localizes more towards the internal part of the centriole cylinder. The Plk4 distribution detected accords with previous findings indicating that the N-terminal part of Cep152 directly interacts with the cryptic polo-box domain of Plk4. (**J**) Colocalization of centriolar proteins is contemporaneous with onset of centriole formation at the 64-cell stage. Centrin2 (left, green), Sas6 (middle, green), and CP110 (right, green) localize to spindle poles only after the 64-cell stage. α-tubulin (red); DNA (blue). Sas6 (green) is not detected until the 32-64-cell stage. Despite the presence of Plk4, centrioles may not be able to form in the mouse embryo until after the 32-cell blastocyst stage because some key proteins for centriole formation are rate limiting and others inappropriately localized.

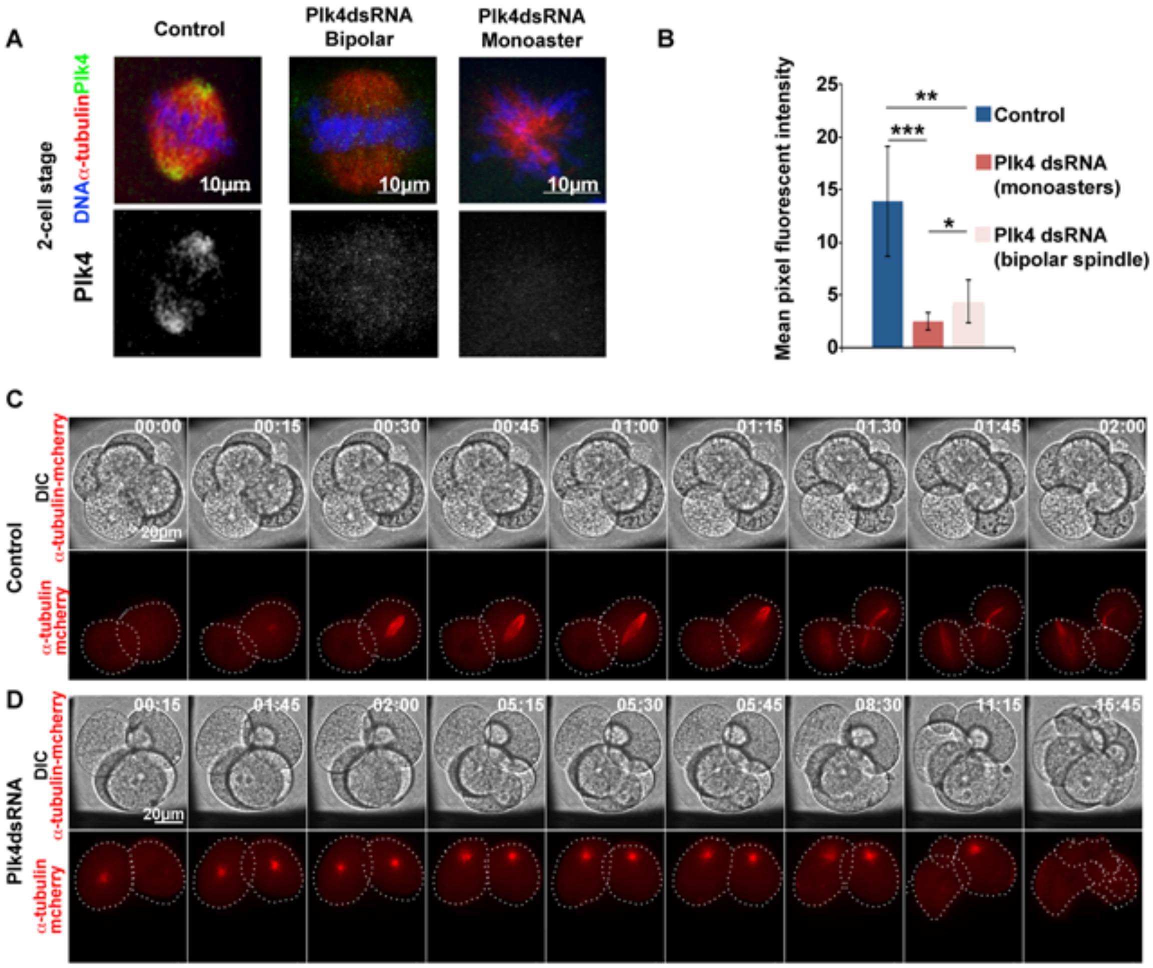

Figure S2, related to Figure2. Low levels of Plk4 results in monopolar spindle formation in the early mouse embryo. (**A-B**) Depletion of Plk4 by injection of dsPlk4 RNA in 2-cell stage embryos. Immunofluorescence image of mitotic figures of control (right) or Plk4 depleted by dsRNA (middle and left) 2-cell embryos. Plk4 (green, and monochrome); α-tubulin (red); DNA (blue). In contrast to control embryos, Plk4 is not observed concentrated to the spindle poles following Plk4 dsRNA injection if bipolar spindle eventually forms. Plk4 dsRNA results in down-regulation of Plk4 protein levels at the time of the first division after injection (**B**). Quantification of the mean pixel intensity for Plk4 signal in control uninjected (n=12) and Plk4dsRNA depleted (n=10) 2-cell stage embryos showing monoasters as well as bipolar spindle (n=11). Embryos were fixed after entering mitosis (8-10 h after injection). The differences of Plk4 mean pixel intensity quantified between the three different groups are significant (***p<0.001; **p<0.005; *p<0.05). Error bars indicate the standard deviation of the average. The reduction of Plk4 levels in Plk4dsRNA depleted blastomeres with monoasters or with bipolar spindles compared to control 2 cell- embryos is 82% and 68%, respectively. (**C-D**) Requirement for Plk4 in bipolar spindle formation. **(C)** Control 4-cell stage embryo in which one blastomere was injected at the 2-cell stage with mRNA for α–tubulin-mcherry. Division is completed within 2h of mitotic entry. (**D**) 4-cell stage embryo in which one blastomere was injected with Plk4 dsRNA at the 2-cell stage. MTs are visualized as in (**C**). Monoasters are formed and mitosis persists for over 10h before cells undergo monopolar cytokinesis. See also Movie S2.

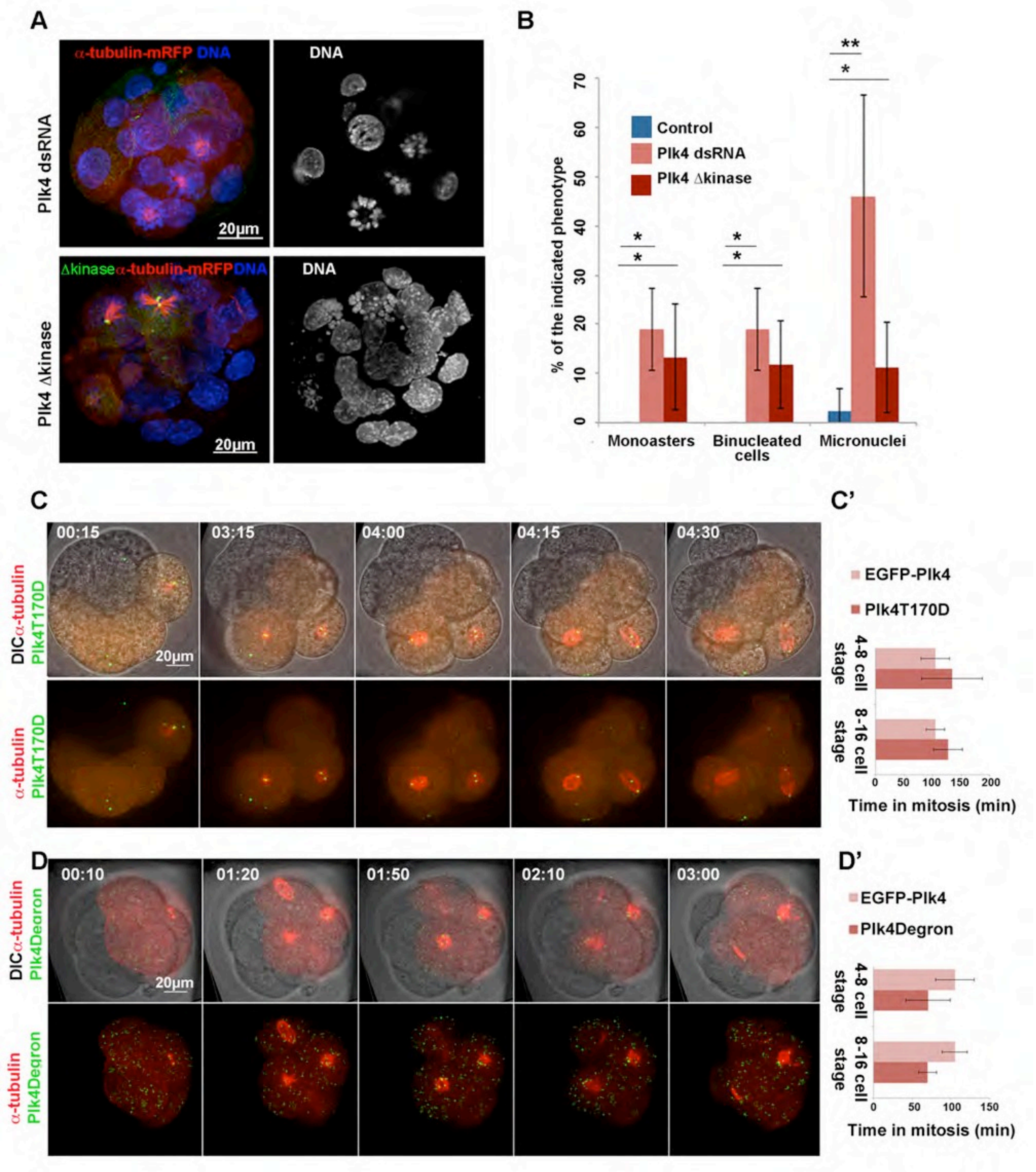

Figure S3 related to Figure 4. Constitutively active or degron mutants of Plk4 do not affect bipolar spindle formation in the early mouse embryo. (A) Monoastral spindles seen in all cells following loss of Plk4 function: Shown here are 16-32 cell embryos that had been injected in a single blastomere at the 2-cell stage with Plk4 dsRNA (upper panels, N=25) or a Δkinase-Plk4 construct comprising only the C-terminal, Polo-box domain of the molecule (lower panels , N=12). Monoasters were detected in both cases. (**B**) Quantification of indicated mitotic defects observed in the embryos in (**A**). The differences quantified between

control embryos and Plk4 dsRNA depleted or Δkinase-Plk4 over-expressing embryos are significant (**$p<0.001$; *$p<0.01$). Error bars indicate the standard deviation of the average. (**C**) Time-lapse series of 4 cell embryos co-injected with mRNA encoding constitutively active Plk4 (green) with α-tubulin-mcherry (red) (N=27, 92% show no phenotype) and quantification of time spent in mitosis (**C'**). Time is shown in hh:mm. Plk4T170D localizes to the spindle poles during mitosis and discrete dots can be observed in the cytoplasm during both interphase and mitosis. Error bars indicate the standard deviation of the average. (**D**)Time-lapse series following co-injection of mRNA encoding a degron mutant form of Plk4 (green) with α–tubulin mcherry (red) (N=26, 84% show no phenotype) and quantification of time spent in mitosis (**D'**). The error bars indicate the standard deviation to the average. See also Movie S5.

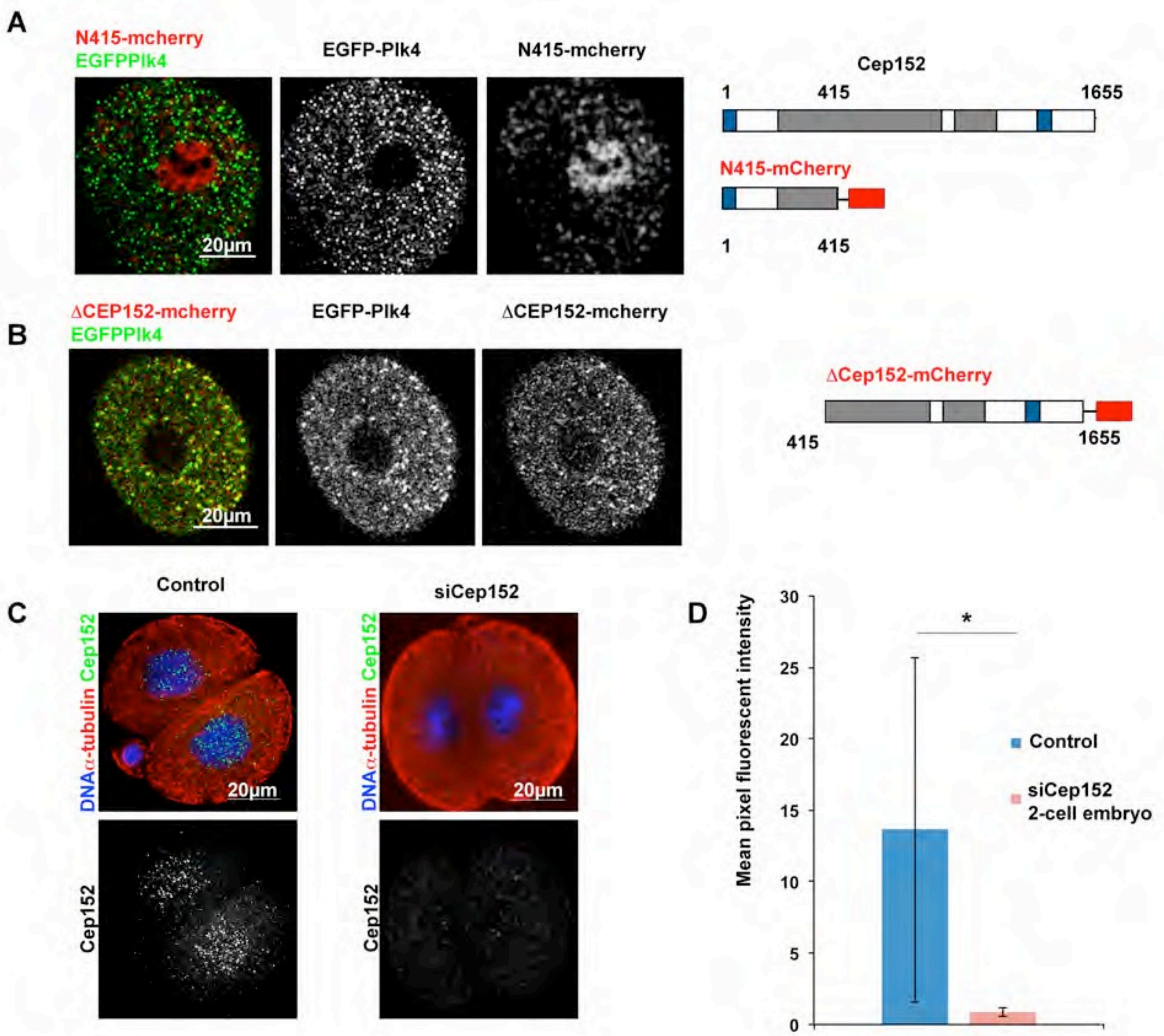

Figure S4 related to Figure 5. (**A**) Selected still of a blastomere expressing EGFP-Plk4 (green and monochrome) and N415-mcherry (red and monochrome). On the right, schematic of 1-415 Cep152 peptide fluorescently tagged with mcherry. N415 -mcherry (1-415aa) is preferentially localized in the nucleus, unlike (**B**) ΔCep152-mcherry (416-1655) schematic on right), which localizes to cytoplasm. (**C**) Depletion of Cep152 following injection of siCep152 RNAs in 2-cell stage embryos. Immunofluorescence image of interphase of control (right) or siCep152 depleted 2-cell embryos (left). Cep152 (green, and monochrome); α-tubulin (red); DNA (blue). siCep152 RNA was co- injected with mRNA encoding α-tubulin-mcherry to monitor injection and embryos were fixed for immunofluorescence 6 hours after injection. Cep152 localization to the nucleus is reduced in the embryos injected with siCep152. (**D**) Cep152 siRNA results in down-regulation of protein levels 6-8 hours after injection. Quantification of the mean pixel intensity for Cep152 signal in control uninjected (n=10) and siCep152 depleted blastomeres (n=12). The difference of Cep152 mean pixel intensity in the nucleus quantified between the three different groups is significant (*$p<0.05$). All the error bars in this figure indicate the standard deviation of the average. The reduction of Cep152 levels in depleted blastomeres compared to control is 94%.

Supplemental data

Movie S1 that relates to Figure 1. Plk4 associates with acentriolar MTOCs of the early mouse embryo. Time lapse imaging of zygotes expressing α-tubulin-mcherry (red) and Plk4-GFP (green). *Z*-stacks are composed of 20 optical sections covering 60 µm and were acquired every 15min. NEBD corresponds to time 00:00 (hh:mm).

Movie S2 that relates to Figure 2. Plk4 depletion results in monopolar spindle formation in the early mouse embryo

Time lapse imaging of 2-4-cell stage transition.

Control: One of the blastomeres was injected with mRNA encoding EGFP-Map4 (green) and Histone H2B-mRFP (red) to follow the dynamic of MT nucleation and spindle assembly at the same time as chromosome condensation and sister chromatid segregation. Below the merged channels for EGFP-Map4 and Histone H2B-mRFP, and above the same channels merged with DIC (black and white).

Plk4 RNAi example 1: For Plk4 depletion blastomeres were coinjected with Plk4 dsRNA, together with mRNA encoding EGFP-Map4 (green) and Histone H2B-mRFP (red). MT nucleation activity from MTOCs is observed at NEBD but no bipolar spindle is formed. The blastomere eventually exits M-phase without undergoing cytokinesis. Below, the merged channels for EGFP-Map4 and Histone H2B-mRFP, and on the top the same channels merged with DIC (black and white).

Plk4 RNAi example 2: In this example for Plk4 depletion, MT nucleation is observed over the chromosomes without the formation of a bipolar spindle. The blastomere exits M- phase without undergoing a normal cytokinesis. Z stacks are composed of 20 optical sections 3 µm apart. Images were acquired every 10min. NEBD corresponds to time 00:00 (hh:mm).

Time lapse imaging of 4-8-cell stage transition.

Control: One of the blastomeres was injected with mRNA encoding α-tubulin-mcherry, to follow the dynamics of spindle assembly (below). Above, DIC images at the corresponding time points. The embryos were imaged during the 4-8 cell stage transition.

Plk4 RNAi: This movie is followed by another time lapse imaging of a 4-cell

embryo in which one of the blastomeres was depleted for Plk4 by injection of Plk4 dsRNA at the 2- cell stage. The embryo was co-injected with mRNA encoding α-tubulin-mcherry, to follow the dynamics of spindle assembly (bottom). DIC images of the embryo collected at the corresponding time points (top). The embryos were imaged from the 4-8 cell stage. See also Figure S2.

Time lapse imaging of 8-16-cell stage transition.

Plk4 RNAi: One of the blastomeres was depleted for Plk4 by injection of Plk4 dsRNA at the 2-cell stage. The embryo was co-injected with mRNA encoding α-tubulin-mcherry, to follow the dynamics of spindle assembly (left). DIC images of the embryo collected at the corresponding time points (top).

Human Plk4-GFP rescue: Time lapse imaging of an 8-cell embryo in which one of the blastomeres was depleted for Plk4 by injection of Plk4 dsRNA at 2-cell stage. The embryo was co-injected with mRNA encoding α-tubulin-mcherry and humanPlk4-GFP, to determine whether the human Plk4 could rescue the Plk4 dsRNA depletion. Below, the α-tubulin-mcherry channel (red) and humanPlk4-GFP (green). Above, DIC images of the embryo merged with α-tubulin-mcherry and humanPlk4-GFP channels. These previous embryos were imaged during the 8-16 cell transition. Z stacks are composed of 20 optical sections 3 μm apart. Images were acquired every 15min. NEBD corresponds to time 00:00 (hh:mm).

Movie S3 relating to Figure 3. Plk4 depletion dramatically reduces kinetics of microtubule regrowth. On the left side, time lapse imaging of the 2-4 cell transition. One of the blastomeres was injected with mRNA encoding humanPlk4-mcherry (red) together with EGFP-EB3 (inverted black) at 2-cell stage. When the first MT nucleation at MTOCS was observed, embryos were subjected to cold treatment. MT regrowth occurs around Plk4 foci surrounding chromosomes. On the right side, time lapse imaging of a 4-cell embryo. At the 2-cell stage, one of the blastomeres was depleted for Plk4 by injection of Plk4 dsRNA. The blastomere was co-injected with mRNA encoding EGFP-EB3 (inverted black). After cold treatment, during regrowth assays, density of MTs was reduced. Z stacks are composed of 4 optical sections 1μm apart. Images were acquired every 3sec. NEBD correspond to time 00:00 (mm:ss).

Movie S4 relating to Figure 4. Expression of kinase defective Plk4 results in monopolar spindle formation in the early mouse embryo.

Time lapse imaging of a 4-cell embryo in which one of the blastomeres was injected with mRNA encoding EGFP-ΔkinasePlk4 together with α-tubulin-mcherry at the 2-cell stage. Below, α-tubulin-mcherry (red) and EGFP-ΔkinasePlk4 (green). Above, DIC images of the embryo merged with α-tubulin-mcherry and EGFP-ΔkinasePlk4 channels. The lower cell completes mitosis and cell division; a monoastral spindle is formed in the upper cell. Time lapse imaging of a 4-cell embryo in which one of the blastomeres was injected with mRNA encoding EGFP-T170APlk4 together with α–tubulin-mcherry at the 2-cell stage. Below, the α-tubulin-mcherry (red) and EGFP-T170APlk4 (green) channels. Above, DIC images of the embryo merged with α-tubulin-mcherry and EGFP-T170APlk4 channels. The embryos were imaged during the 4-8 cell transition. Z stacks are composed of 20 optical sections 3 μm apart and images were acquired every 15min. NEBD corresponds to time 00:00 (hh:mm).

Movie S5, related to Figure 4 and Figure S3. Constitutively active or degron mutants of Plk4 do not affect spindle formation in the early mouse embryo.

Time lapse imaging of a 4-cell embryo in which one of the blastomeres was co-injected with mRNA encoding a constitutively active form of Plk4 with a mutation at the T-loop, T170DPlk4, and mRNA for α-tubulin-mcherry at the 2-cell stage. α-tubulin-mcherry (red) and EGFP-T170DPlk4 (green) channels are shown. This movie is followed by a time lapse imaging of a 4-cell embryo in which one of the blastomeres was injected with mRNA encoding a non-degradable, degron-mutant form of Plk4, EGFP-DegronPlk4 together with α-tubulin-mcherry at the 2-cell stage. α-tubulin-mcherry (red) and EGFP-DegronPlk4 (green) channels are shown. The embryos were imaged during the 4-8 cell transition. Z stacks are composed of 20 optical sections 3 μm apart. Images were acquired every 15min. NEBD corresponds to time 00:00 (hh:mm).

Movie S6 that is related to Figure 5. Depletion of Cep152 results in monopolar spindle formation in the early mouse embryo. On the left, time lapse imaging of a 2-cell embryo in which one of the blastomeres was injected

with mRNA encoding EGFP-Map4 (green) and Histone H2B-mRFP (red) to follow the dynamic of MT nucleation and spindle assembly, simultaneously with chromosome condensation and sister chromatid segregation. Below, the merged channels for EGFP- Map4 and Histone H2B-mRFP. Above, the corresponding channels merged with DIC. On the right, time lapse imaging of a 2-cell embryo in which one of the blastomeres was depleted for Cep152 by siRNA and co-injected with mRNAs encoding EGFP-Map4 (green) and Histone H2B-mRFP (red). Below the merged channels for EGFP-Map4 and Histone H2B-mRFP. Above, the corresponding channels merged with DIC. The embryos were imaged at the 2-4 cell stage. Z stacks are composed of 20 optical sections 3 μm apart. Images were acquired every 10min. NEBD corresponds to time 00:00 (hh:mm).

Movie S7 relating to Figure 6. Membrane targeted Cep152 directs active, but not inactive, Plk4 to this ectopic site to rescue double depletion of endogenous Plk4 and Cep152. On the left, time lapse imaging of a 2-cell embryo in which one of the blastomeres was depleted for Cep152 by siRNA and for Plk4 by dsRNAi. The embryo was co-injected with mRNA encoding CD8GFP-humanCep152 (green) and humanPlk4-mcherry (red) to determine whether these constructs could rescue Plk4 and Cep152 depletion. EGFP-EB3 mRNA (green) was also co-injected to visualize MT nucleation. Bipolar spindles are formed with their poles in proximity to the membrane and blastomeres are able to divide. On the right, time lapse imaging of a 2-cell embryo in which one of the blastomeres was depleted for Cep152 by siRNA, and for Plk4 by dsRNAi. The embryo was co-injected with mRNA encoding CD8GFP-humanCep152 (green) and the kinase dead humanPlk4D159A-mcherry (red). MT nucleation was observed by expression of EGFP- EB3 mRNA (green), which was co-injected. Although MT nucleation is observed, bipolar spindle does not assemble and cytokinesis fails. The embryos were imaged during the 2-4 cell stage. Z stacks are composed of 20 optical sections 3 μm apart. Images were acquired every 15min. NEBD corresponds to time 00:00 (hh:mm). This first set of movies is followed by substacks showing colocalizing CD8GFP-humanCep152 (green) and humanPlk4 wild-type (red, on the left) or the kinase dead human Plk4D159A (red, on the right) and identified and tracked through mitosis. A movement between membrane and spindle poles is observed if wild-type human -Plk4 is injected. On

the contrary, Kinase dead humanPlk4D159A localizes around the MT nucleation sites, undergoing random movement.

Table S1- Sequence of oligonucleotides

T7Plk4_300	5'TAATACGACTCACTATAGGGATGGCGGCGTGCATCGGGGAGAGGATCGAG3'
SP6Plk4_870	'5'ATTTAGGTGACACTATAGAAGTCCATGTGCACTTCGAGTTGCAATTTCTG3'
T170AFOR	5'GAATATGCCACATGAAAAGCACTATGCACTCTGTGGGACTCCTAATTATATTTC3'
T170AREV	5'GAAATATAATTAGGAGTCCCACAGAGTGCATAGTGCTTTTCATGTGGCATATTC3'
T170DFOR	5'GAATATGCCACATGAAAAGCACTATGACCTCTGTGGGACTCCTAATTATATTTC3'
T170DREV	5'GAAATATAATTAGGAGTCCCACAGAGGTCATAGTGCTTTTCATGTGGCATATTC3'
DegronFOR	5'GAGGACTCAATGGATGCTGGGCATGCTGCACTTTCCACAACAATTACAGC3';
DegronREV	'5'GCTGTAATTGTTGTGGAAAGTGCAGCATGCCCAGCATCCATTGAGTCCTC3'
D159AFor	'5'CGTAATATGAACATCAAGATTGCTGCTTTTGGGCTGGCAACTCAACTGAAAATGCC3'
D159Rev	5'GGCATTTTCAGTTGAGTTGCCAGCCCAAAAGCAGCAATCTTGATGTTCATATTACG3'.
mPLK4_For-	5'GGGGACAAGTTTGTACAAAAAAGCAGGCTTCATGGTTGGTCAACCACTTCCAAATAAAATTACTGTA3'
mPLK4_Rev	5'GGGGACCACTTTGTACAAGAAAGCTGGGTCTTATGATAAGGCTTTAGGTGAACTAGTATTACCACG3'

Sequence of the oligonucleotides used as primers in the PCRs described in Experimental Procedures. Each primer (left column) is indicated in the description of different experiments.

Supplemental References

Habedanck, R., Stierhof, Y.-D., Wilkinson, C.J., and Nigg, E.A. (2005). The Polo kinase Plk4 functions in centriole duplication. Nature Cell Biology *7*, 1140–1146.
