# Supplementary figures and images for: Spindle Formation in the Mouse Embryo Requires Plk4 in the Absence of Centrioles
Source: Dev Cell. 2013 Dec 9;27(5):586–97. doi: 10.1016/j.devcel.2013.09.029 (PMC3898710; doi:10.1016/j.devcel.2013.09.029)

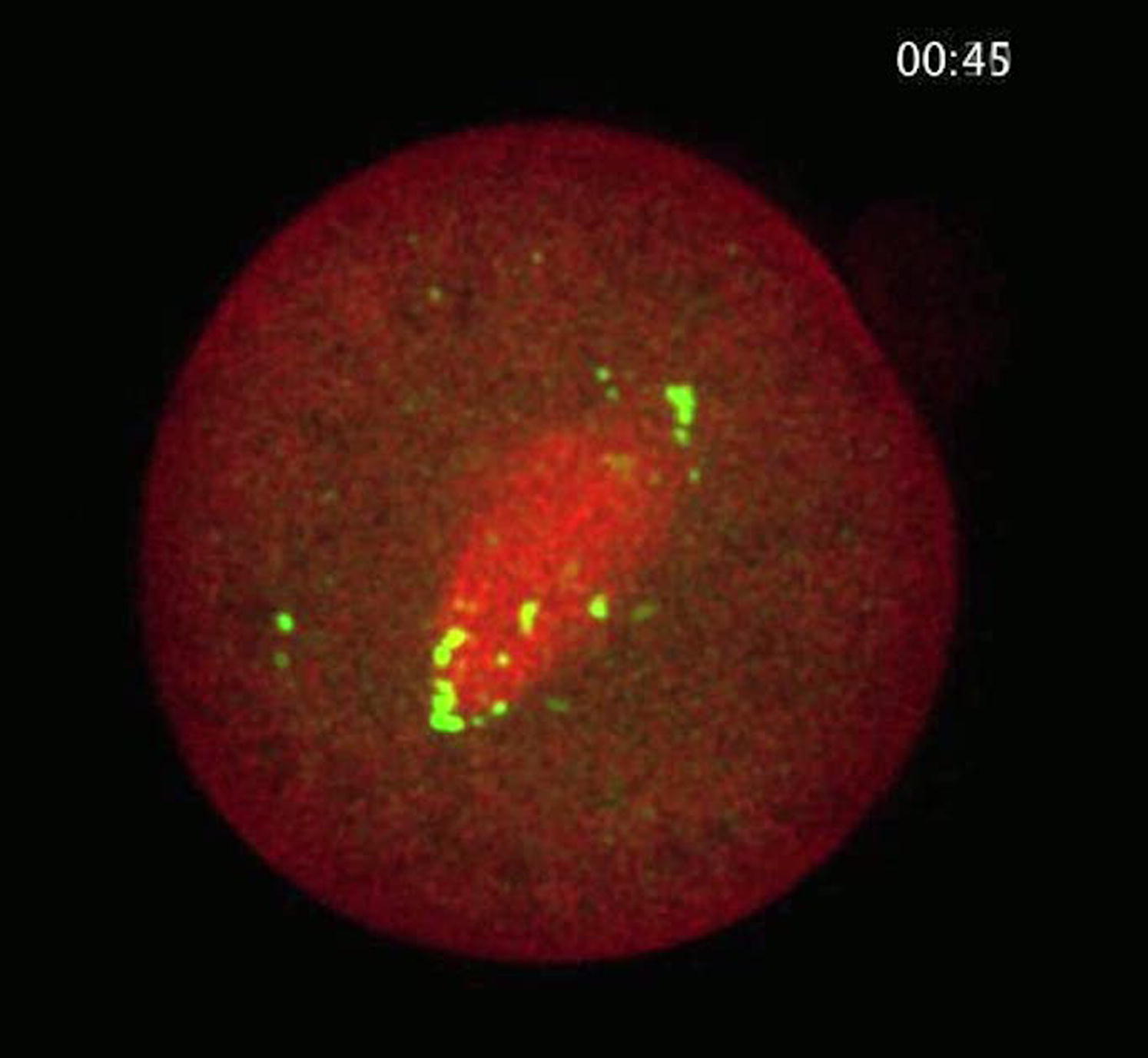

Supplement: Movie S1. Plk4 Associates with Acentriolar MTOCs of the Early Mouse Embryo, Related to Figure 1 — Time-lapse imaging of zygotes expressing α-tubulin-mcherry (red) and Plk4-GFP (green). Z stacks are composed of 20 optical sections covering 60 μm and were acquired every 15 min. NEBD corresponds to time 00:00 (hr:min). [file mmc2.jpg]

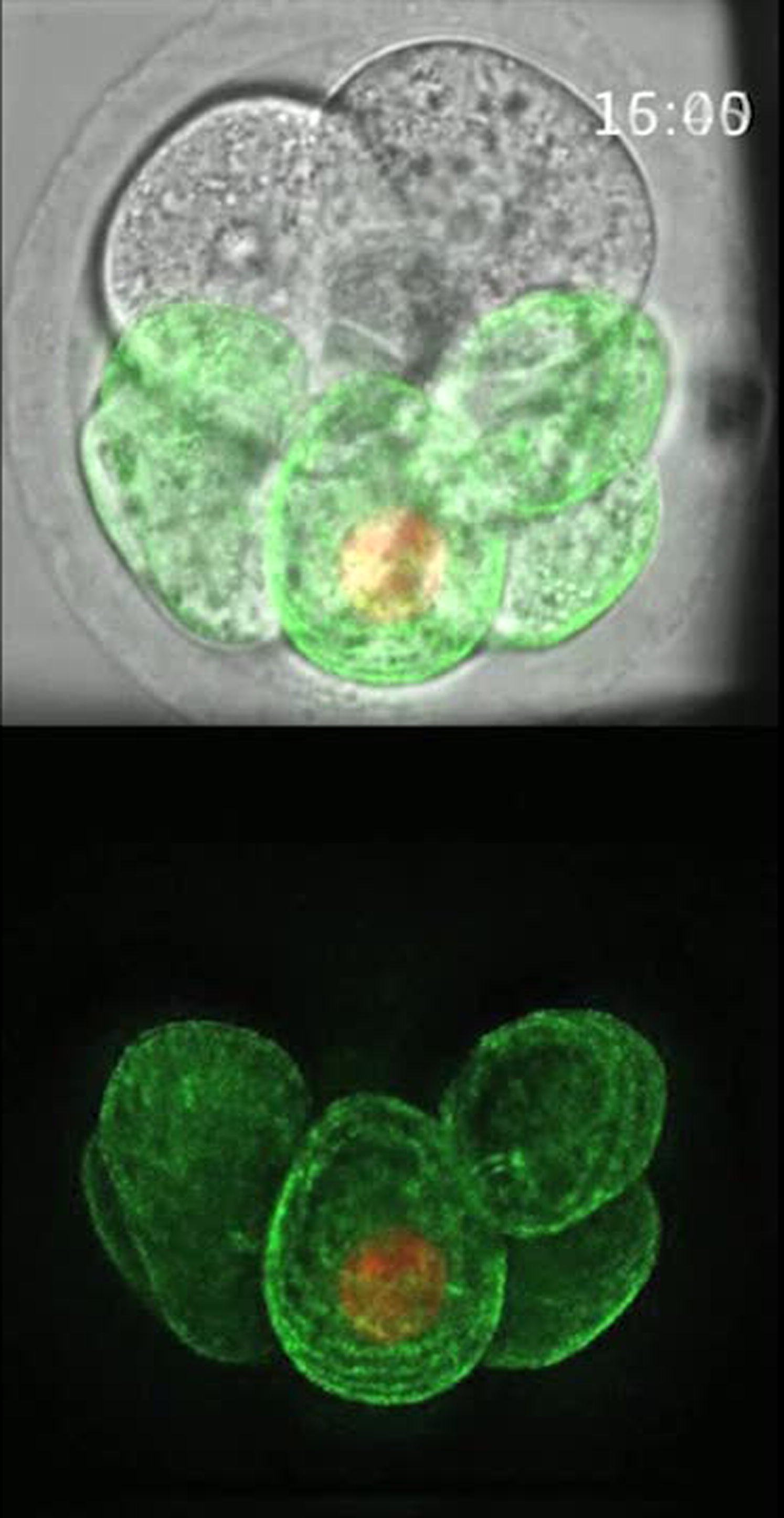

Supplement: Movie S2. Plk4 Depletion Results in Monopolar Spindle Formation in the Early Mouse Embryo, Related to Figure 2 — Time-lapse imaging of 2- to 4-cell stage transition. Control: One of the blastomeres was injected with mRNA encoding EGFP-MAP4 (green) and histone H2B-mRFP (red) to follow the dynamic of MT nucleation and spindle assembly at the same time as chromosome condensation and sister chromatid segregation. Below are the merged channels for EGFP-MAP4 and histone H2B-mRFP, and above are the same channels merged with DIC (black and white). Plk4 RNAi example 1: For Plk4 depletion, the blastomere was coinjected with Plk4 dsRNA, together with mRNA encoding EGFP-MAP4 (green) and histone H2B-mRFP (red). MT nucleation activity from MTOCs is observed at NEBD, but no bipolar spindle is formed. The blastomere eventually exits M phase without undergoing cytokinesis. Below are the merged channels for EGFP-MAP4 and histone H2B-mRFP, and on the top are the same channels merged with DIC (black and white). Plk4 RNAi example 2: In this example for Plk4 depletion, MT nucleation is observed over the chromosomes without the formation of a bipolar spindle. The blastomere exits M phase without undergoing normal cytokinesis. Z stacks are composed of 20 optical sections 3 μm apart. Images were acquired every 10 min. NEBD corresponds to time 00:00 (hr:min). Time-lapse imaging of 4- to 8-cell stage transition. Control: One of the blastomeres was injected with mRNA encoding α-tubulin-mcherry to follow the dynamics of spindle assembly (below). Above are DIC images at the corresponding time points. The embryos were imaged during the 4- to 8-cell stage transition. Plk4 RNAi: This movie is followed by another time-lapse imaging of a 4-cell embryo in which one of the blastomeres was depleted for Plk4 by injection of Plk4 dsRNA at the 2-cell stage. The embryo was coinjected with mRNA encoding α-tubulin-mcherry to follow the dynamics of spindle assembly (bottom). DIC images of the embryo collected at the corresponding time points are shown on top. The embryos were imaged from the 4- to the 8-cell stage. See a [file mmc3.jpg]

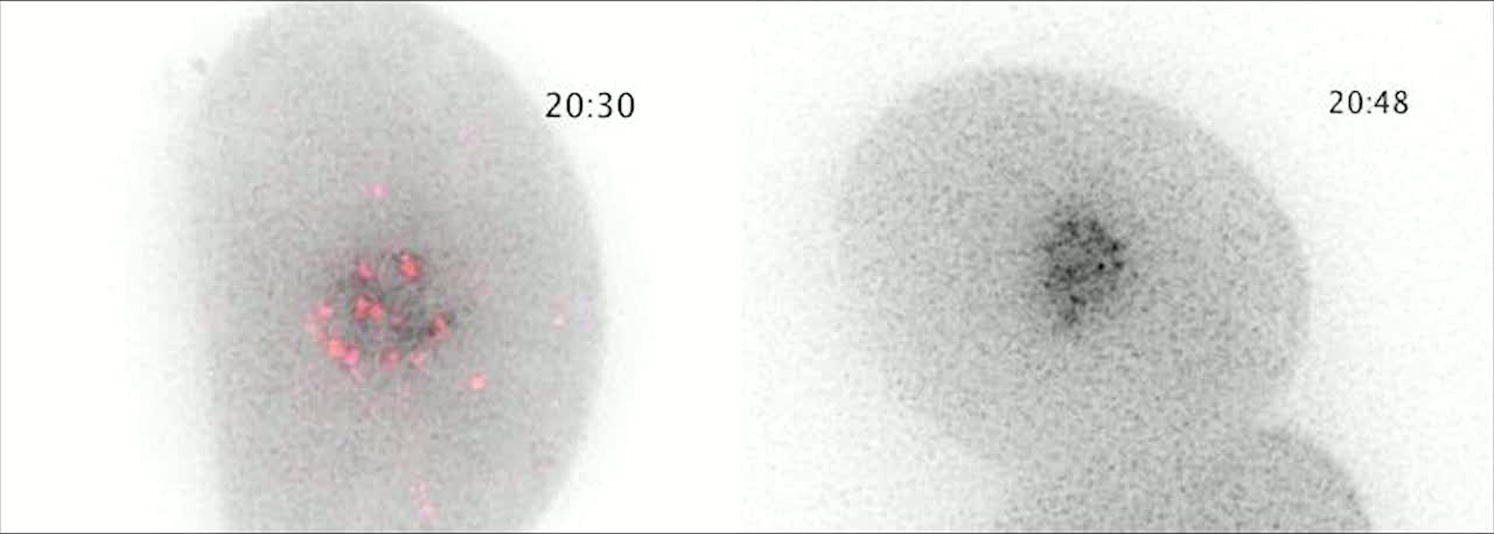

Supplement: Movie S3. Plk4 Depletion Dramatically Reduces Kinetics of Microtubule Regrowth, Related to Figure 3 — On the left side is time-lapse imaging of the 2- to 4-cell transition. One of the blastomeres was injected with mRNA codifying humanPlk4-mcherry (red) together with EGFP-EB3 (inverted black) at 2-cell stage. When the first MT nucleation at MTOCs was observed, embryos were subjected to cold treatment. MT regrowth occurs around PLK4 foci around chromosomes. On the right side is time-lapse imaging of a 4-cell embryo. At the 2-cell stage, one of the blastomeres was depleted for Plk4 by injection of Plk4 dsRNA. The blastomere was coinjected with mRNA codifying EGFP-EB3 (inverted black). After cold treatment, during regrowth assays, density of MTs was reduced. Z stacks are composed of four optical sections 1 μm apart. Images were acquired every 3 s. NEBD correspond to time 00:00 (min:s). [file mmc4.jpg]

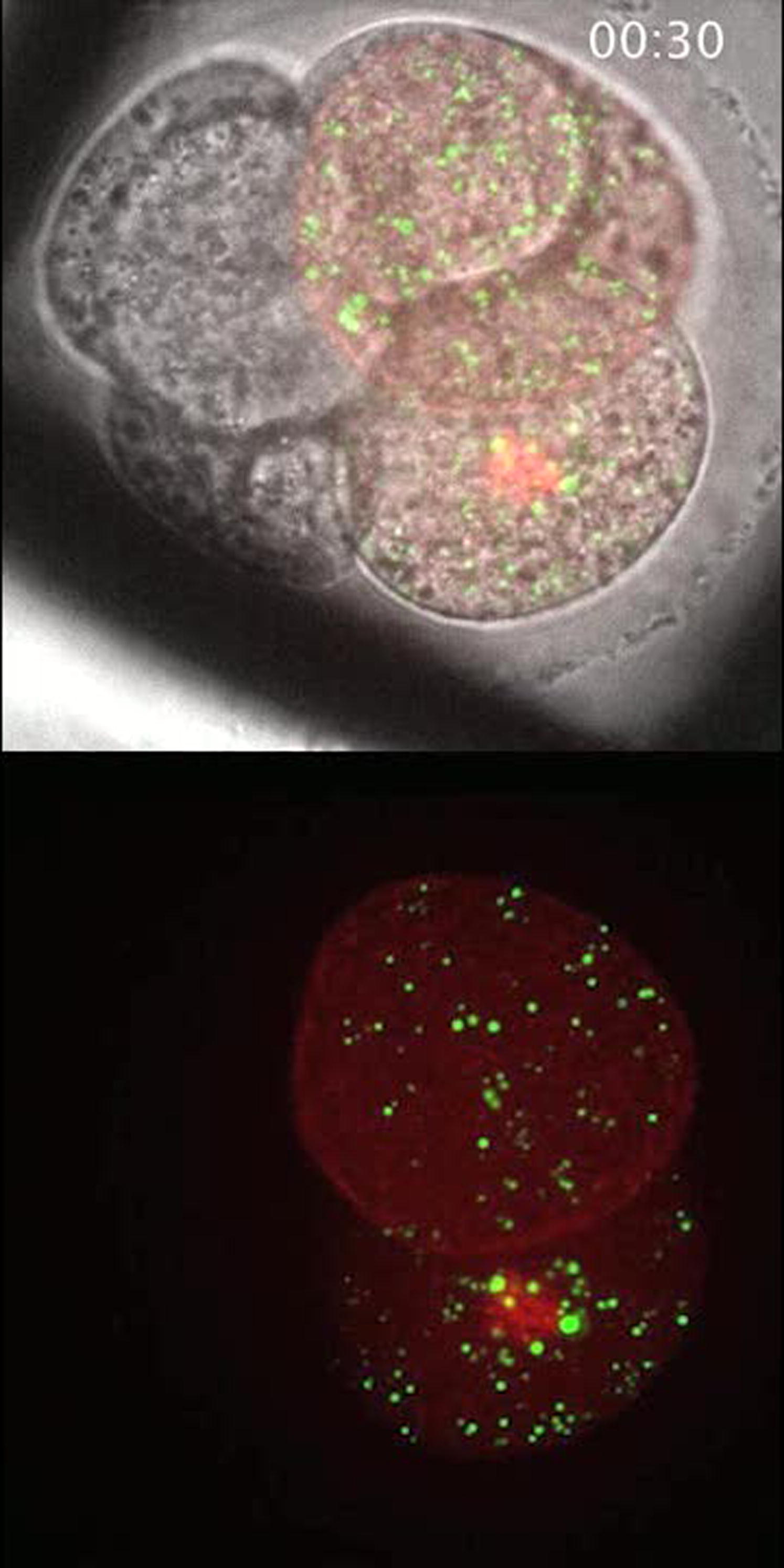

Supplement: Movie S4. Expression of Kinase-Defective Plk4 Results in Monopolar Spindle Formation in the Early Mouse Embryo, Related to Figure 4 — Time-lapse imaging of a 4-cell embryo in which one of the blastomeres was injected with mRNA encoding EGFP-ΔkinasePlk4 together with α-tubulin-mcherry at the 2-cell stage. Below are α-tubulin-mcherry (red) and EGFP-ΔkinasePlk4 (green). Above are DIC images of the embryo merged with α-tubulin-mcherry and EGFP-ΔkinasePlk4 channels. The lower cell completes mitosis and cell division; a monoastral spindle is formed in the upper cell. Time-lapse imaging of a 4-cell embryo in which one of the blastomeres was injected with mRNA codifying EGFP-T170APlk4 together with α-tubulin-mcherry at the 2-cell stage. Below are the α-tubulin-mcherry (red) and EGFP-T170APlk4 (green) channels. Above are DIC images of the embryo merged with α-tubulin-mcherry and EGFP-T170APlk4 channels. The embryos were imaged during the 4- to 8-cell transition. Z stacks are composed of 20 optical sections 3 μm apart, and images were acquired every 15 min. NEBD corresponds to time 00:00 (hr:min). [file mmc5.jpg]

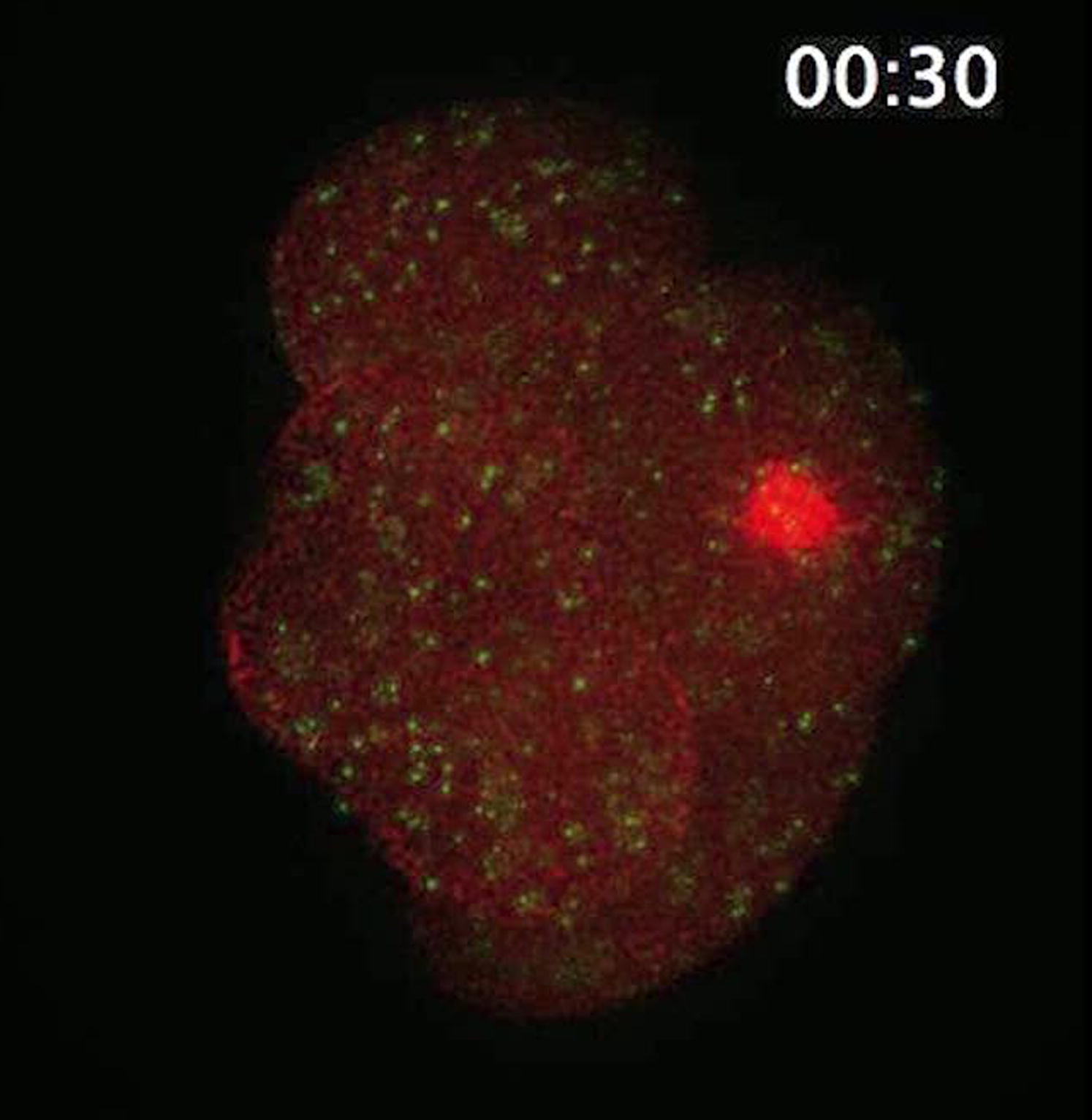

Supplement: Movie S5. Constitutively Active or Degron Mutants of Plk4 Do Not Affect Spindle Formation in the Early Mouse Embryo, Related to Figure 4 — Time-lapse imaging of a 4-cell embryo in which one of the blastomeres was coinjected with mRNA encoding a constitutively active form of PLK4 with a mutation at the T loop, T170DPlk4, and mRNA for α-tubulin-mcherry at the 2-cell stage. α-tubulin-mcherry (red) and EGFP-T170DPlk4 (green) channels are shown. This movie is followed by a time-lapse imaging of a 4-cell embryo in which one of the blastomeres was injected with mRNA encoding a nondegradable, degron-mutant form of PLK4, EGFP-DegronPlk4 together with α-tubulin-mcherry at the 2-cell stage. α-tubulin-mcherry (red) and EGFP-DegronPlk4 (green) channels are shown. The embryos were imaged during the 4- to 8-cell transition. Z stacks are composed of 20 optical sections 3 μm apart. Images were acquired every 15 min. NEBD corresponds to time 00:00 (hr:min). [file mmc6.jpg]

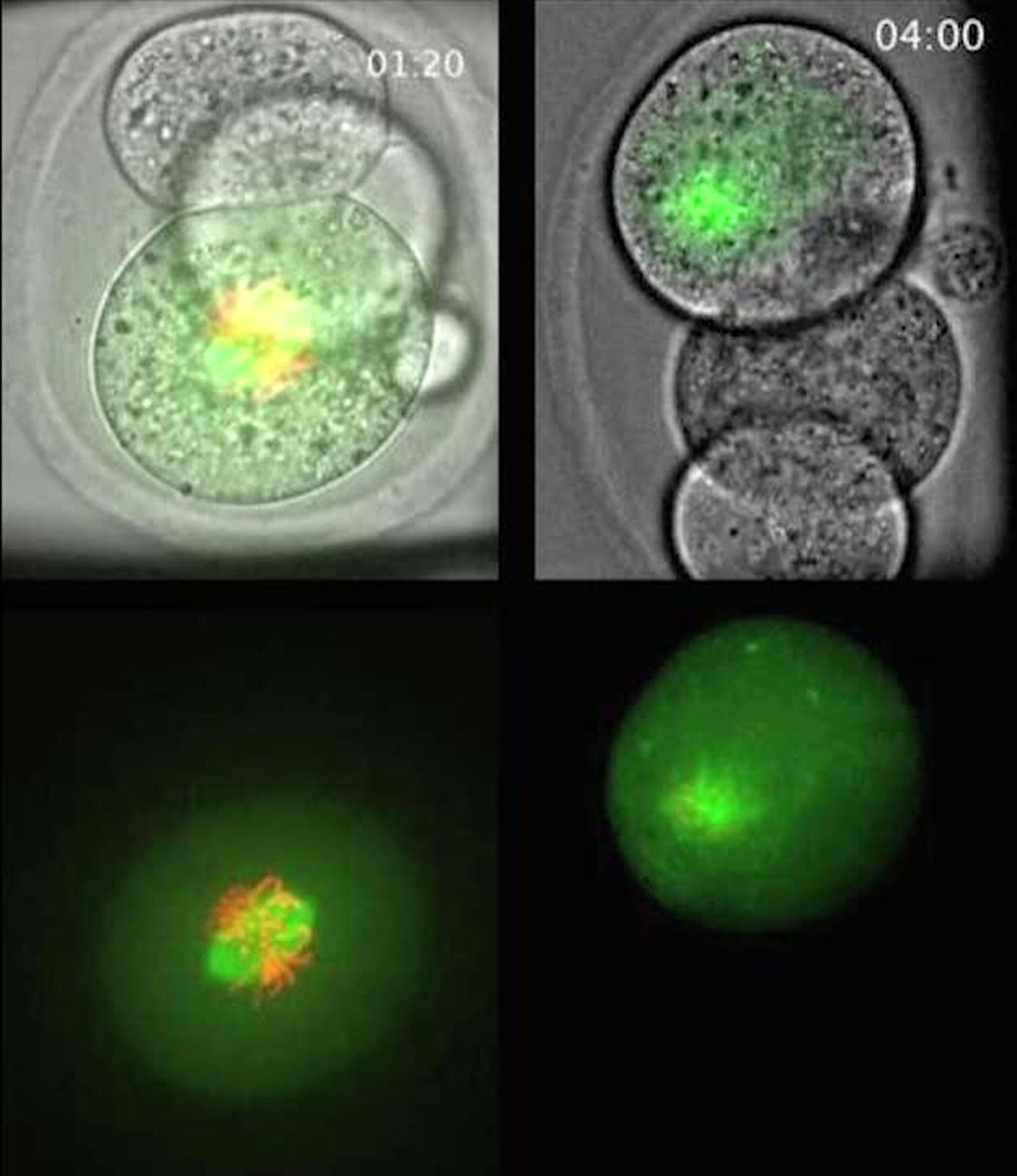

Supplement: Movie S6. Depletion of Cep152 Results in Monopolar Spindle Formation in the Early Mouse Embryo, Related to Figure 5 — On the left is time-lapse imaging of a 2-cell embryo in which one of the blastomeres was injected with mRNA encoding EGFP-MAP4 (green) and histone H2B-mRFP (red) to follow the dynamic of MT nucleation and spindle assembly simultaneously with chromosome condensation and sister chromatid segregation. Below are the merged channels for EGFPMAP4 and histone H2B-mRFP. Above are the corresponding channels merged with DIC. On the right is time-lapse imaging of a 2-cell embryo in which one of the blastomeres was depleted for Cep152 by siRNA and coinjected with mRNAs encoding EGFP-MAP4 (green) and histone H2B-mRFP (red). Below are the merged channels for EGFP-MAP4 and histone H2B-mRFP. Above are the corresponding channels merged with DIC. The embryos were imaged at the 2- to 4-cell stage. Z stacks are composed of 20 optical sections 3 μm apart. Images were acquired every 10 min. NEBD corresponds to time 00:00 (hr:min). [file mmc7.jpg]

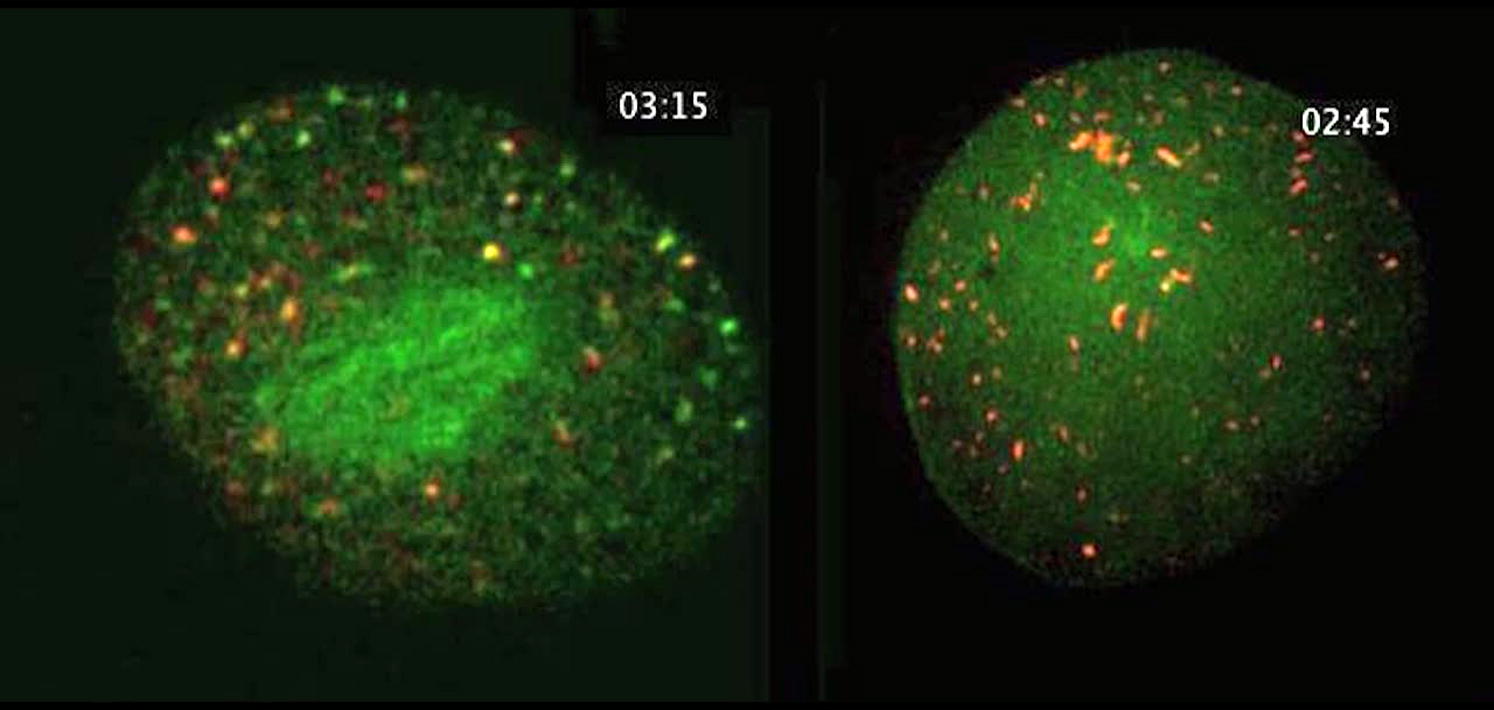

Supplement: Movie S7. Membrane-Targeted Cep152 Directs Active, but Not Inactive, Plk4 to This Ectopic Site to Rescue Double Depletion of Endogenous Plk4 and Cep152, Related to Figure 6 — On the left is time-lapse imaging of a 2-cell embryo in which one of the blastomeres was depleted for Cep152 by siRNA and for PLK4 by dsRNAi. The embryo was coinjected with mRNA encoding CD8GFP-humanCep152 (green) and humanPLK4-mcherry (red) to determine whether these constructs could rescue Plk4 and Cep152 depletion. EGFP-EB3 mRNA (green) was also coinjected to visualize MT nucleation. Bipolar spindles are formed with their poles in proximity to the membrane, and blastomeres are able to divide. On the right is time-lapse imaging of a 2-cell embryo in which one of the blastomeres was depleted for Cep152 by siRNA and for PLK4 by dsRNAi. The embryo was coinjected with mRNA encoding CD8GFP-humanCep152 (green) and the kinase dead humanPLK4D159A-mcherry (red). MT nucleation was observed by expression of EGFPEB3 mRNA (green), which was coinjected. Although MT nucleation is observed, bipolar spindle does not assemble and cytokinesis fails. The embryos were imaged during the 2- to 4-cell stage. Z stacks are composed of 20 optical sections 3 μm apart. Images were acquired every 15 min. NEBD corresponds to time 00:00 (hr:min). This first set of movies is followed by substacks showing colocalizing CD8GFP-Cep152 (green) and humanPLK4 wild-type (red, on the left) or the kinase dead Plk4D159A (red, on the right) identified and tracked through mitosis. A movement between membrane and spindle poles is observed if wild-type human Plk4 is injected. Conversely, kinase-dead PLK4D159A localizes around the MT nucleation sites, undergoing random movement. [file mmc8.jpg]
